# Real Time Spectroscopic Ellipsometry Analysis of First Stage CuIn_1−*x*_Ga_*x*_Se_2_ Growth: Indium-Gallium Selenide Co-Evaporation

**DOI:** 10.3390/ma11010145

**Published:** 2018-01-16

**Authors:** Puja Pradhan, Puruswottam Aryal, Dinesh Attygalle, Abdel-Rahman Ibdah, Prakash Koirala, Jian Li, Khagendra P. Bhandari, Geethika K. Liyanage, Randy J. Ellingson, Michael J. Heben, Sylvain Marsillac, Robert W. Collins, Nikolas J. Podraza

**Affiliations:** 1 Center for Photovoltaics Innovation and Commercialization & Department of Physics and Astronomy, University of Toledo, Toledo, OH 43606, USA; puja.pradhan@rockets.utoledo.edu (P.P.); puruswottam@hotmail.com (P.A.); dattyga@gmail.com (D.A.); aibdah@rockets.utoledo.edu (A.-R.I.); prakash.koirala@utoledo.edu (P.K.); jian.li2@rockets.utoledo.edu (J.L.); khagendra.bhandari@utoledo.edu (K.P.B.); Geethika.Liyanage@rockets.utoledo.edu (G.K.L.); Randy.Ellingson@utoledo.edu (R.J.E.); Michael.Heben@utoledo.edu (M.J.H.); robert.collins@utoledo.edu (R.W.C.); 2 Virginia Institute of Photovoltaics, Old Dominion University, Norfolk, VA 23529, USA; smarsill@odu.edu

**Keywords:** spectroscopic ellipsometry, III_2_-VI_3_ semiconductor materials, real time analysis, complex dielectric function, photovoltaic cells, thickness measurement, compositional analysis, CuIn_1−*x*_Ga_*x*_Se_2_

## Abstract

Real time spectroscopic ellipsometry (RTSE) has been applied for in-situ monitoring of the first stage of copper indium-gallium diselenide (CIGS) thin film deposition by the three-stage co-evaporation process used for fabrication of high efficiency thin film photovoltaic (PV) devices. The first stage entails the growth of indium-gallium selenide (In_1−*x*_Ga*_x_*)_2_Se_3_ (IGS) on a substrate of Mo-coated soda lime glass maintained at a temperature of 400 °C. This is a critical stage of CIGS deposition because a large fraction of the final film thickness is deposited, and as a result precise compositional control is desired in order to achieve the optimum performance of the resulting CIGS solar cell. RTSE is sensitive to monolayer level film growth processes and can provide accurate measurements of bulk and surface roughness layer thicknesses. These in turn enable accurate measurements of the bulk layer optical response in the form of the complex dielectric function ε = ε_1_ − iε_2_, spectra. Here, RTSE has been used to obtain the (ε_1_, ε_2_) spectra at the measurement temperature of 400 °C for IGS thin films of different Ga contents (*x*) deduced from different ranges of accumulated bulk layer thickness during the deposition process. Applying an analytical expression in common for each of the (ε_1_, ε_2_) spectra of these IGS films, oscillator parameters have been obtained in the best fits and these parameters in turn have been fitted with polynomials in *x*. From the resulting database of polynomial coefficients, the (ε_1_, ε_2_) spectra can be generated for any composition of IGS from the single parameter, *x*. The results have served as an RTSE fingerprint for IGS composition and have provided further structural information beyond simply thicknesses, for example information related to film density and grain size. The deduced IGS structural evolution and the (ε_1_, ε_2_) spectra have been interpreted as well in relation to observations from scanning electron microscopy, X-ray diffractometry and energy-dispersive X-ray spectroscopy profiling analyses. Overall the structural, optical and compositional analysis possible by RTSE has assisted in understanding the growth and properties of three stage CIGS absorbers for solar cells and shows future promise for enhancing cell performance through monitoring and control.

## 1. Introduction

Photovoltaics (PV) technology based on the crystalline forms of silicon (Si) was the first to be commercialized for manufacturing on a large scale and continues to be the most widely practiced technology to date [1]. In spite of its widespread application, Si is not an ideal material for solar cells because of its indirect bandgap and thus low absorption coefficient over wide regions within the solar spectrum. As a result, a relatively large volume of crystalline Si is required for complete absorption of solar irradiance with above-bandgap (>1.1 eV) photon energies. A promising broad strategy for reducing materials costs in PV manufacturing is focused on the advancement of thin film technologies [2]. In a thin film PV technology, a layer of strongly-absorbing, high-quality semiconductor material is deposited onto an inexpensive coated substrate such as rigid glass, flexible steel sheets, or polymer foil. Suitably strong absorption in a thin film semiconductor can be obtained via either an amorphous material or a direct bandgap polycrystalline material. Hydrogenated amorphous silicon (a-Si:H) was one of the first thin film materials to be explored in depth because of its much higher absorption coefficient at visible wavelengths compared to crystalline Si. For an amorphous solid, this higher absorption coefficient is due to the relaxation of crystal momentum conservation. In addition to a-Si:H, a large number of direct bandgap polycrystalline binary, ternary and quaternary compound semiconductors has been investigated over the last several decades as possible solar cell absorber layers [2]. Rapid progress in the field of thin film PV materials has been made over the last three years through investigations of the organic-inorganic perovskite halide absorbers [3]. In fact, all such thin film materials have been researched intensively due to their potential as lower cost alternatives to Si wafer technology once the performance limitations of the thin films could be overcome [1,2,3].

Considering the thin film PV technologies extensively commercialized to date, those based on absorbers of polycrystalline copper indium-gallium diselenide (CuIn_1−*x*_Ga_*x*_Se_2_; CIGS), an alloy of chalcopyrite compounds, and polycrystalline CdTe, a zinc blende compound, exhibit the highest laboratory performance [4]. The improvement of CIGS solar cells has accelerated in recent years and a laboratory-scale record conversion efficiency of 22.6% has been achieved in this technology [5]. Full scale commercial CIGS modules with efficiencies greater than 15% have been developed on flexible stainless-steel substrates [6], a manufacturing technology that enables the use of high-throughput, roll-to-roll thin film deposition methods. The CIGS compound alloy has many advantages as a solar cell absorber including its high absorption coefficient made possible by a direct bandgap, its bandgap tunability and associated profiling capabilities made possible by variability in the atomic ratio *x* = [Ga]/{[In] + [Ga]} and controllable p-type doping made possible by a reduction in the Cu content below 25 at % resulting in Cu vacancies which serve as shallow acceptors [7,8]. The highest performing CIGS solar cells are fabricated with thickness-averaged Ga compositions in the range of 0.2 to 0.4 having bandgaps in the range of 1.1 to 1.3 eV [7,8,9,10]. The application of alkaline post deposition treatments has been one of the important recent directions for improvement in the record efficiencies of CIGS solar cells [5,11,12]. Another important direction is the reduction of optical losses at the top contact [13]. The bandgap adjustability of the CIGS material system also enables attractive options for tandem devices. For example, the CIS or CIGS device with an absorber bandgap in the range of 1.0 to 1.1 eV is suitable as the bottom cell in combination with a top cell having a perovskite absorber with a wider bandgap of ~1.6–1.7 eV [14,15]. An efficiency of 10.9% has been reported recently for such a CIGS/perovskite tandem device [15]. 

Among the various methods that have been developed over the years for fabricating CIGS absorbers, three-stage co-evaporation is the process that has led to the record efficiency solar cells on the laboratory scale [5,16,17,18]. For co-evaporated CIGS absorber films, the concentration of defects in the material is strongly influenced by the reaction pathway and substrate temperature during film growth [19]. Co-evaporation in three distinct stages enables greater control and optimization of the crystalline grain structure, the Cu composition, the defect concentrations and the bandgap profile throughout the thickness of the resulting CIGS absorber. In each stage of the conventional three-stage process for the CIGS absorber, an individual subset of the four elements of Cu, In, Ga and Se is deposited by co-evaporation on Mo-coated soda lime glass at an elevated temperature. These stages include an initial In+Ga+Se deposition at ~400 °C (stage I), a Cu+Se deposition at ~500–600 °C (stage II) and a final In+Ga+Se deposition at ~500–600 °C (stage III). Because ~65% or more of the final CIGS absorber layer thickness is generated in stage I of the three-stage process, it is critical to ensure the desired composition of (In_1−x_Ga_x_)_2_Se_3_ (IGS) in this stage and, thus, the ultimate composition profile throughout the absorber thickness after all three stages are complete. As a result, precise control of the IGS composition, as well as control of the final thickness, are desirable in stage I in order to obtain a CIGS absorber layer that optimizes the device performance. Optimization is possible in the cell’s open circuit voltage (V_oc_) via bandgap profiling and in its fill factor (FF) via grain growth enhancement. Hence, in order to understand the nature of the three-stage co-evaporation process and to optimize this process, it is necessary to first investigate the growth evolution and resulting properties of the precursor IGS layer. Given the continuous nature of the CIGS growth process, this analysis must be performed in real time during stage I and in-situ after stage I termination, in the latter case before the start of stage II.

Real time and in-situ spectroscopic ellipsometry (SE) serve as effective tools for analysis of the optical properties of individual solar cell materials and the multilayer structures of complicated thin film devices [20,21]. For example, in a study relevant to the present research, Ranjan et al. have presented the complex dielectric function (ε = ε_1_ − iε_2_) spectra of CIGS obtained by SE measurements as a function of the Cu content in the film. These SE measurements were performed in-situ after (In_1−x_Ga_x_)_2_Se_3_ (IGS) exposure to Cu and Se co-evaporants for different stage II durations at the stage II temperature of 570 °C [22]. In the present research, real time spectroscopic ellipsometry (RTSE) has been applied to study stage I of CIGS fabrication. In this stage of deposition IGS thin films have been deposited by co-evaporation of In, Ga and Se with different intended Ga compositions *x* on Mo surfaces at a substrate temperature of 400 °C. RTSE has been used to obtain the structural evolution and (ε_1_, ε_2_) spectra for IGS thin films of different values of *x*, as measured by energy-dispersive X-ray spectroscopy (EDS), and over different ranges of bulk layer thickness accumulated during the depositions. In the study of the dependence of (ε_1_, ε_2_) on *x*, substrates have been employed that differ from those optimized for solar cells. For this first study, the IGS films were fabricated on 5000 Å thick Mo films which were deposited in turn on thermally oxidized Si wafers, rather than the soda lime glass (SLG) of the solar cell structures. For this alteration from standard device processing, IGS deposition occurs on a smoother underlying Mo surface for enhanced sensitivity to the structural evolution and the (ε_1_, ε_2_) spectra of IGS. In the second study concerning the thickness dependence, the focus is on a single IGS stage I deposition with *x* = 0.30. For this IGS deposition, the underlying Mo layer was 8000 Å thick, as in the optimized solar cell structure. The resulting IGS layer was converted to CIGS by performing stages II and III in sequence for integration of the layer into a solar cell. 

Through the variation in (ε_1_, ε_2_) with photon energy, it is possible to extract information on the IGS alloy composition, the relative void content and the grain size or defect density in the film. Toward this goal, IGS (ε_1_, ε_2_) spectra have been fitted using an analytical expression that includes the sum of two oscillator terms, one associated with transitions between parabolic bands modeled as a critical point (CP) oscillator and the other associated with broader background transitions modeled as a Tauc-Lorentz (TL) oscillator. The photon energy-independent oscillator parameters have been expressed first in terms of Ga content *x* for films of similar bulk layer thickness and second in terms of bulk layer thickness d_b_ for the IGS deposition with *x* = 0.30. Hence, the variations of the oscillator parameters with Ga content *x* have been obtained that enable determination of the (ε_1_, ε_2_) spectra for an IGS alloy within a specified thickness range for any value of *x*. In addition to a compositional analysis capability afforded by the (ε_1_, ε_2_) spectra, the deduced structural evolution can assist in understanding the complete three-stage CIGS deposition process, optimizing the IGS for conversion to CIGS and generally in monitoring and controlling the solar cell fabrication.

## 2. Experimental Details

Each IGS deposition was performed on a Mo surface by co-evaporation using nominally fixed fluxes of In, Ga and Se at a substrate temperature of 400 °C. Routine analysis of film compositions was performed ex-situ after deposition using energy-dispersive X-ray spectroscopy (EDS) with an electron microscope (Hitachi TM1000, Hitachi High Technologies in America, Schaumburg, IL, USA). The EDS attachment to the microscope was manufactured by Oxford Instruments, Abingdon, UK. Two types of experiments were performed for analysis of the (ε_1_, ε_2_) spectra of the IGS films as described in the following paragraphs. In these experiments which focus on materials and device structures, efforts were taken to reduce the surface roughness thickness forming on the IGS layer by depositing the IGS on Mo film surfaces with reduced roughness layer thicknesses. In general, smoother underlying Mo surfaces lead to smoother IGS films. With smoother IGS films, more accurate structural and optical models of the IGS films can be developed when interpreting RTSE data acquired for different alloy compositions and over different ranges of bulk layer thickness. 

In order to explore the effect of alloy composition on the (ε_1_, ε_2_) spectra for a fixed bulk layer thickness range, a series of IGS thin films with different Ga contents *x* = [Ga]/{[In] + [Ga]} = 0.00 (In_2_Se_3_), 0.10, 0.25, 0.31, 0.37, 0.45, 0.56, 0.69 and 1.00 (Ga_2_Se_3_) was investigated. These films were deposited on Mo surfaces so as to simulate the stage I CIGS process and were measured by RTSE throughout the total accumulated ~9000 Å thickness. This final thickness would result in a 1.4 μm thick CIGS layer if the subsequent two stages were to be completed. The substrate structures were Si wafers 2.5 cm × 7.6 cm in size with ~250 Å thermally grown SiO_2_, in turn coated with optically opaque Mo. The Mo thin films were deposited by direct current (DC) magnetron sputtering using a high purity Mo target (99.9999%) in a high purity Ar environment (99.9999%). The DC power supply for sputtering was set at 150 W, with an Ar pressure of ~4 mTorr and an Ar flow of 30 sccm. The Mo films were deposited at room temperature to a thickness of 5000 Å. The intent of a thinner Mo layer in this case, relative to the ~8000 Å thick layer deposited in optimized PV devices, was to reduce the surface roughness on the Mo while providing the same surface chemistry as that used in three-stage deposition of CIGS for solar cells. 

In order to explore the evolution of the (ε_1_, ε_2_) spectra with thickness at the fixed Ga ratio of *x* = 0.30, RTSE data were collected during the ~1.7 μm thick stage I deposition of a CIGS absorber with an ultimate thickness of ~2.5 μm. This absorber was incorporated into a solar cell using the standard three stage co-evaporation process. In this study, a substrate structure was used consisting of Mo-coated SLG, as previously optimized for devices. In fact, a maximum efficiency of 17.4% was obtained for the CIGS solar cells fabricated in this process, which includes final deposition of a 910 Å thick MgF_2_ anti-reflection coating by electron beam evaporation. For this optimized substrate structure, the Mo was sputtered as a bilayer to a thickness of 8000 Å on SLG held at 250 °C. A relatively thin Mo layer (~1000 Å) is deposited as the first layer of the bilayer using a high Ar pressure of ~15 mTorr at a sputtering power of 150 W with an Ar flow of 140 sccm. Then, a thicker layer (~7000 Å) is deposited at a lower Ar pressure of ~4 mTorr at a sputtering power of 150 W with an Ar flow of 30 sccm. The elevated Mo substrate temperature of 250 °C for the 8000 Å thick bilayer served to reduce the surface roughness layer thickness on the Mo film leading to smoother surface for the over-deposited IGS as well. The RTSE measurements were performed in-situ during IGS film growth in order to characterize the evolution of the bulk and surface roughness layer thicknesses, as well as the variations in the (ε_1_, ε_2_) spectra as functions of composition and accumulated IGS thickness. The ability of SE and RTSE to extract accurate relative values of the surface roughness layer thickness on amorphous and polycrystalline semiconductor and metallic thin films has been demonstrated in reports that present correlations of the SE and RTSE analysis results with atomic force microscopy [23,24,25]. Because of the real-time nature of the measurements, the (ε_1_, ε_2_) spectra of the IGS films are characteristic of the 400 °C deposition temperature. As a result, these spectra are applicable for routine monitoring of the high temperature stage I process by RTSE. A commercially-available rotating-compensator multichannel spectroscopic ellipsometer with a spectral range of 0.74–6.5 eV (M-2000 DI, J.A. Woollam Co., Lincoln, NE, USA) was used for the RTSE measurements. The angle of incidence for RTSE was fixed within the range from 70.24° to 70.67° with typical confidence limits of ±0.17°. For the RTSE measurements, the beam is 2 mm in diameter and illuminates the sample surface forming an ellipse with a major axis length of 6 mm. The beam strikes the center of the substrate/film where the variation of *x* = [Ga]/{[In} + [Ga]} over the beam area is no more than ±0.005 and the variation in thickness is no more than 1%, as estimated from detailed mapping studies of CIGS depositions [26]. Thus, uniformity of the optical properties and thicknesses over the beam area is assumed in all RTSE analyses. The complete ellipsometric spectra (ψ, Δ) are collected in real time in 0.25 s during the IGS film growth process. No more than 4 Å of IGS film thickness is deposited in this time. The time interval between two successive spectral acquisitions in (ψ, Δ) is selected as ~0.4 s such that no more than a few monolayers of the film are deposited on the substrate during the interval. Data analysis was performed using software provided with the instrument (WVASE version 3.888 and CompleteEASE version 5.20). 

Complex dielectric functions of the IGS layers in parametric form were established by least squares regression analysis of results obtained by inversion using fixed structural parameters and a fixed complex dielectric function for the underlying Mo [20]. The parametric form for IGS used here includes three terms,

ε = ε_1,∞_ + ε_CP_(*E*) + ε_TL_(*E*), (1)

where ε_1,∞_ is an energy-independent contribution to the real part of the dielectric function, ε_CP_(*E*) represents a single critical point (CP) oscillator and ε_TL_(*E*) represents a single broad Tauc-Lorentz (TL) background oscillator. The mathematical form of the complex component describing the CP oscillator is given by:

(2) $$\varepsilon_{\mathrm{CP}}\left(E\right)=A\Gamma^{\mu }e^{i\varphi }/{\left(2E_{g}-2E-i\Gamma \right)}^{\mu }$$

with variable parameters of amplitude, energy, broadening, phase, and exponent {*A*, *E_g_*, *Γ*, ϕ, µ}, respectively. This equation is based on the assumption that in the neighborhood of a critical point, the bands are parabolic, and the momentum matrix element is independent of photon energy. The mathematical form of the imaginary part of the TL oscillator is given by

(3) $$\varepsilon_{2,\mathrm{TL}}\left(E\right)=\{\begin{array}{c} \frac{AE_{0}\Gamma \;{\left(E-E_{g}\right)}^{2}}{{\left(E^{2}-E_{0}\right)}^{2}+\Gamma^{2}E^{2}}.\frac{1}{E}\;\;\;\;,\;E>E_{g}\;;\\ \;\;\;\;\;0\;\;\;\;\;,E\leq E_{g}\;. \end{array}$$

The four free parameters in this expression include the amplitude (*A*), resonance energy (*E*_0_), broadening (*Γ*) and bandgap (*E*_g_). ε_1,TL_(*E*) is deduced from ε_2,TL_(*E*) through the Kramers-Kronig relationship.

**Figure 1:**
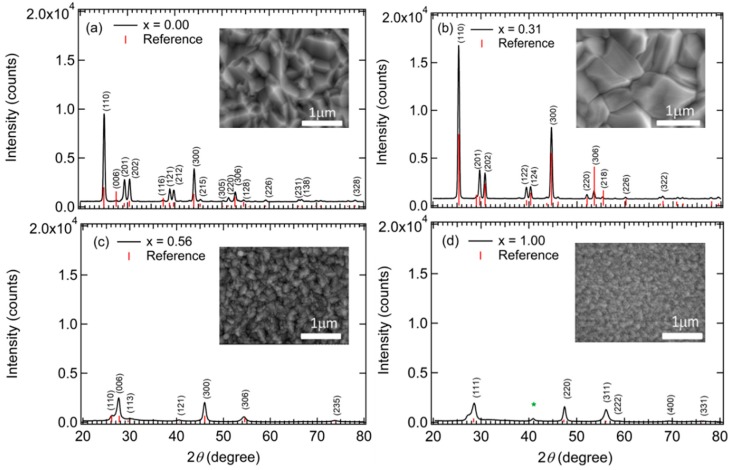
X-ray diffraction (XRD) patterns for ~9000 Å thick IGS films of different alloy composition values *x*. The XRD patterns are consistent with expectations for (In_1−x_Ga_x_)_2_Se_3_ obtained from MDI JADE: (**a**) PDF # 97-064-0476; (**b**) PDF # 97-063-4424; (**c**) PDF # 97-009-2068 and (**d**) PDF # 97-007-6754. The scanning electron microscopy (SEM) images for the same IGS films are shown in the insets. These samples from the composition series were also studied by real time spectroscopic ellipsometry (RTSE) as described in Section 3.1.

In addition to the in-situ RTSE studies, ex-situ analysis techniques were applied as well for the study of the IGS composition series. These techniques include not only EDS but also X-ray diffractometry (XRD), scanning electron microscopy (SEM) and transmission electron microscopy (TEM). The results of EDS measurements are summarized in Table 1. These results show an (In + Ga):Se ratio ranging from 0.66 to 0.84 with an average value near 0.75 for the nine depositions. Thus, the IGS films are Se poor relative to the 0.67 stoichiometric ratio, suggesting the possible existence of In_1−x_Ga_x_Se phases [27]. XRD patterns and SEM images for selected IGS films are shown in Figure 1. XRD patterns of IGS films prepared at substrate temperatures similar to those of the precursor films in stage I CIGS fabrication have been presented and discussed in detail in several literature reports [27,28,29,30,31,32,33,34,35]. The diffractions obtained for IGS films with *x* = 0.00 and 0.31 in Figure 1 are consistent with the γ-phase of (In_1−x_Ga_x_)_2_Se_3_ [27,32] but without additional phases, which suggests that the films studied here exhibit a hexagonal (defect-wurtzite) crystal structure over this range of *x*. The hexagonal crystal structure is also maintained for *x* = 0.56, although some peaks observed for *x* = 0.00 and 0.31 are missing for the *x* = 0.56 sample. In contrast for the film with *x* = 1.00, the observed peak positions are consistent with those given in the literature for the cubic (disordered zinc blende) α-phase of Ga_2_Se_3_ [28,31,33,35]. The diffractions observed for the Ga-rich films are not very strong, which suggests small grain sizes or highly disordered nanocrystalline materials, as also evidenced by the SEM images. For *x* = 1.00, one feature can be observed at 2θ ~ 41° in the XRD pattern (*) that does not correspond to the α-phase or any other phase of Ga-Se. This feature may be attributed to a small fraction of elemental Se on the surface or in grain boundary regions of the Ga_2_Se_3_ film. The XRD results in Figure 1 for Ga_2_Se_3_ are similar to those observed previously for films deposited by metalorganic chemical vapor deposition at 450 °C to 500 °C, whereas films deposited at higher temperature exhibit stronger and sharper diffraction peaks [33]. Finally, the SEM images in Figure 1 suggest an increase in grain size for hexagonal IGS from *x* = 0.00 to 0.31 which is consistent with the observed enhancement in the diffractions. For the film with *x* = 0.56, however, grain sizes are much smaller than those of the two films deposited with lower *x*. This structural transition apparently between *x* = 0.31 and 0.56 occurs without a change from the hexagonal crystal structure. For cubic Ga_2_Se_3_ (*x* = 1.00) the grain sizes from SEM are even smaller still, again consistent with the XRD observations of the broader diffractions.

**Figure 2:**
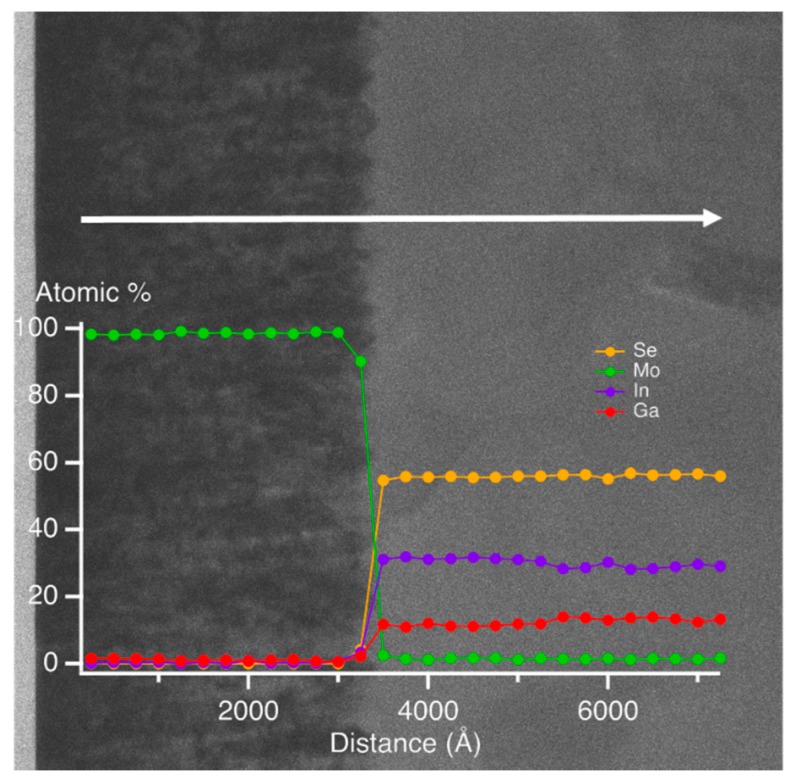
Cross-sectional transmission electron microscopy (TEM) image and elemental line profiles by energy-dispersive X-ray spectroscopy (EDS) for a Mo/IGS structure with IGS composition *x* = 0.31 highlighting the interface region. The EDS measurements were performed across the interface every 250 Å in depth.

A cross-sectional TEM image and an EDS elemental line profile highlighting the Mo/IGS interface are shown in Figure 2 for a single IGS film deposited with *x =* 0.31. These measurements were performed using a Hitachi HD-2300A scanning transmission electron microscope equipped with an EDS detector (Hitachi High Technologies in America, Schaumberg, IL, USA). The TEM sample was prepared by Ga focused ion beam (FIB) milling using a FEI Quanta 3D FEG-dual beam microscope (FEI North America NanoPort, Hillsboro, OR, USA). The EDS measurements were performed every 250 Å in the vicinity of the Mo/IGS interface. Figure 2 suggests an abrupt interface with no evidence of a MoSe_2_ layer, at least at the probed location and within the ~200 Å resolution of EDS. The formation mechanism of MoSe_2_ at the Mo/CIGS interface for thin film CIGS fabricated by three-stage co-evaporation has been studied previously by high resolution TEM [36]. In the study of Ref. [36], it was concluded that the lower substrate temperature of 350 °C in the first stage of IGS deposition was not sufficiently high for the IGS layer to react with Mo and that the reaction to form MoSe_2_ occurs during the higher temperature stages II and III. This finding is consistent with the results of Figure 2, as well as the RTSE studies in Section 3. Finally, the TEM image suggests a peak-to-valley roughness thickness of ~200–300 Å at the Mo/IGS interface for this sample.

## 3. RTSE Results and Discussion

The key components of this article presented in Section 3.1 and Section 3.2 below focus on the application of RTSE for determination of the (ε_1_, ε_2_) spectra for IGS thin films of different Ga compositions *x* and thickness ranges, respectively. To ensure relevance of the results to devices, the IGS films explored in the study of the composition dependence in Section 3.1 were deposited on Mo thin film surfaces in processes that simulate stage I of standard three-stage co-evaporation of CIGS. In the study of the thickness dependence in Section 3.2, however, stage I of an actual CIGS solar cell process (rather than a simulation of the process) was measured by RTSE as described previously in Section 2. The strategy for interpreting SE data in general involves extracting (ε_1_, ε_2_) spectra together with the structural properties by increasing the number of data sets analyzed simultaneously so that the number of data points is far larger than the number of deduced values. This approach serves to reduce parameter correlations and to obtain results with greater confidence. 

Following this approach, the RTSE data collected in these studies of IGS deposition were analyzed by focusing simultaneously on a large dataset consisting of multiple pairs of ellipsometric (ψ, Δ) spectra acquired at different times over a selected time range of interest [37,38]. A Kramers-Kronig consistent b-spline function with a 0.2 eV node spacing was applied in this analysis as an optical model for the (ε_1_, ε_2_) spectra from 0.74 to 6.0 eV [39]. From the onset of bulk layer growth to the end of the selected time range for data analysis, the (ε_1_, ε_2_) spectra of the IGS are assumed to be time independent. As a result, a structural model for the IGS film with an assumed uniform bulk layer is applied. Such a single layer model for the bulk layer is also applicable in the rare circumstances that the (ε_1_, ε_2_) spectra of the entire uniform bulk layer, from substrate to ambient, evolve with time as in an annealing process. Most often, however, when a time dependence in the bulk layer (ε_1_, ε_2_) spectra occurs, the dependence is restricted to newly deposited material and an optical property profile is built into the film as it is deposited. Such a profile requires a multilayer or virtual interface model in the analysis of the bulk layer, which then becomes a greater challenge as the overlying surface roughness layer increases in thickness [40]. In addition to the single uniform bulk layer, a surface roughness layer must be included in the structural model for the IGS film. Typically in this study, the void contents in the surface roughness layers are fixed at 50 vol % for surface roughness layer thicknesses <90 Å and are allowed to vary for such thicknesses >90 Å. An unweighted error function is used to evaluate the quality of each overall fit [41], for which the final results are the (ε_1_, ε_2_) spectra of the IGS and the time evolution of the bulk and surface roughness layer thicknesses [38]. 

Thus, for the multi-time RTSE analysis procedure described in the previous paragraph, the number of acquired data points is much larger than the number of unknowns in the model. In fact, assuming *M* time points selected for analysis, as well as *N* spectral points, the number of measured data values is 2*MN*. The number of unknowns is 2*M* + 2*N*, which include 2*N* (ε_1_, ε_2_) values versus photon energy and 2*M* bulk and surface roughness layer thicknesses versus time. Thus, as long as 2*MN* > 2(*M* + *N*), then at least in theory, the assumed fixed (ε_1_, ε_2_) spectra and the two layer thicknesses versus time can be deduced by least squares fitting over the selected time range. Because of the large number of spectral positions (*N* ~ 700), simultaneous Kramers-Kronig consistent b-spline smoothing of the (ε_1_, ε_2_) spectra is performed as part of the analysis. This step reduces the spectral noise, generated for example by the biplate compensators [42] and also reduces the number of free parameters defining the (ε_1_, ε_2_) spectra determined in the analysis. 

After obtaining the (ε_1_, ε_2_) spectra, as defined by its b-spline parameters and the time evolution of the structural parameters for a given sample, the (ψ, Δ) spectra corresponding to the center time of the multiple sets are selected for an additional analysis step. This step consists of fixing the structural parameters and subjecting the associated (ψ, Δ) spectra to numerical inversion in order to determine (ε_1_, ε_2_) spectra appropriate for the center time. In the inversion, of course, no assumptions are made regarding the parametric form of the (ε_1_, ε_2_) spectra. The resulting inverted (ε_1_, ε_2_) spectra, a close representation of the experimental data, are subsequently fitted using a physics-based parametric model that includes CP and TL oscillators describing the IGS bulk layer material. The expressions for (ε_1_, ε_2_) spectra of the CP oscillator and the ε_2_ spectra of the TL oscillator are given previously as Equations (2) and (3), respectively. The reason for the additional analysis step is to advance beyond a b-spline representation of (ε_1_, ε_2_) since the parameters of such a representation do not provide clear insights into the nature of the electronic oscillators that describe the spectra. Also, the inverted (ε_1_, ε_2_) spectra rather than the b-spline spectra are fitted using the oscillator model in this step. This ensures that a closer representation of the experimental data, rather than a fit to those data, are fitted using the oscillator model.

### 3.1. Variation of IGS Complex Dielectric Function with Ga Composition (x)

**Figure 3:**
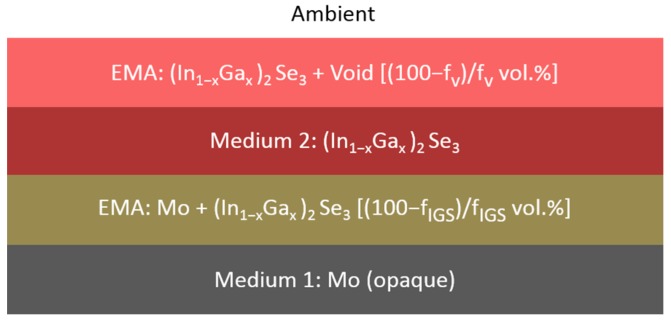
Three-layer structural model used for the analysis of RTSE data acquired in the later stages of the depositions for IGS thin films prepared under conditions that simulate the stage I process of CIGS co-evaporation. In this later stage model, it is assumed that the 50 vol % voids in the surface roughness on the uncoated Mo film are completely filled by the deposited IGS material.

The first set of multi-time analysis procedures was applied to RTSE data collected on IGS films fabricated with different Ga contents, *x*, on Si/SiO_2_/Mo substrates. The goal is to determine simultaneously the structural evolution and the (ε_1_, ε_2_) spectra of the bulk layer after it has stabilized in the later stage of deposition. In this later stage of deposition, it is assumed that the voids in the underlying Mo surface roughness layer have been completely filled by IGS material, yielding an interface roughness layer with the same thickness as the surface roughness layer on the uncoated Mo. The model applied in this analysis consists of five media and three layers, as shown in Figure 3, including (i) an opaque Mo film as a substrate, (ii) the Mo/IGS interface roughness layer arising from the roughness on the Mo surface, (iii) the uniform IGS bulk layer, (iv) the IGS surface roughness layer and (v) an ambient medium of vacuum. For these analyses, the Mo/IGS interface layer thickness was fixed at a value deduced from a measurement of the Mo surface roughness thickness at room temperature prior to substrate heating and deposition, with values being in the 69–88 Å range. After the 50 vol % voids of the interface layer are completely filled by depositing IGS, the contents of the two materials in this interface layer are assumed to be 50/50 vol % Mo/IGS. The (ε_1_, ε_2_) spectra of the interface and surface roughness layers are determined by the Bruggeman effective medium approximation (EMA) as indicated in Figure 3. The Mo/IGS interface roughness thickness of 75 Å, as deduced optically for IGS with composition *x* = 0.31, can be compared to the peak-to-valley result of 200–300 Å from the TEM of Figure 2. A large difference is possible since it has been found that the roughness thickness deduced by SE analysis is 1.0–1.5 times the root mean-square roughness on semiconductor and metal surfaces as obtained by atomic force microscopy [23,25]. 

Table 1 includes the initial and final times, t_i_ and t_f_, defining the multi-time analysis range, and the center time t*, defining the inversion point for the final (ε_1_, ε_2_) determination, associated with each of the depositions yielding IGS of different Ga contents *x*. The bulk layer thickness values d_b_ deduced as the best fit results at t_i_, t* and t_f_ are also given in Table 1 along with the best fit surface roughness layer thickness d_s_ and the fixed surface roughness void content f_v_ both at t*. Finally, in the last two columns of Table 1, the quality of the multi-time best fit, given as the mean square error (MSE), and the instantaneous deposition rate at t*, given as the time derivative of the effective thickness d_eff_, are provided. The effective thickness is generally defined as the product of the thickness and the material volume fraction summed over all layers that incorporate the material. Thus, for IGS, the effective thickness is given by d_eff_ = d_i_ f_IGS_ + d_b_ + d_s_(1 − f_v_), where d_i_ is the Mo/IGS interface thickness and f_IGS_ is its IGS volume fraction as indicated in Figure 3. As can be observed from Table 1, the center time in the analysis was chosen for an approximate bulk layer thickness of 4000 Å. Also, it is observed that at t*, the surface roughness thickness is a maximum for In_2_Se_3_ and a minimum for Ga_2_Se_3_. For In-rich alloys with 0 < *x* ≤ 0.45, the surface roughness thickness lies within the range from 90 to 105 Å and shows no clear trend with *x*, whereas for alloys with *x* ≥ 0.45, a continuous decrease occurs with increasing *x*.

**Figure 4:**
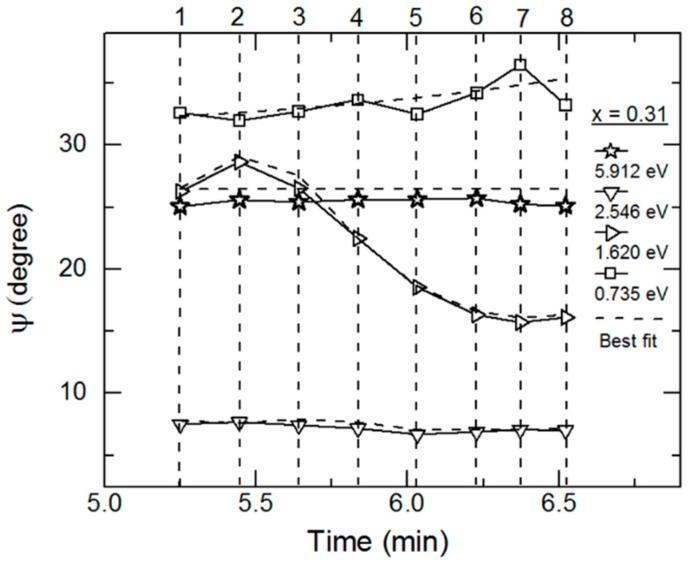
RTSE data (points) collected at the photon energy values of 0.735, 1.620, 2.546 and 5.912 eV for the IGS thin film deposition with *x* = 0.31. Data analysis was performed to extract the time evolution of the film structure and the (ε_1_, ε_2_) spectra of IGS over the analysis range from 0.74 to 6.0 eV. The dashed lines represent the best fit to the data using the structural model of Figure 3 and a Kramers-Kronig consistent b-spline model for the (ε_1_, ε_2_) spectra with a node spacing of 0.2 eV. Structural parameters at the starting, center and ending points are given in Table 1. In such multi-time analyses, the (ε_1_, ε_2_) spectra are assumed to be time independent from the start of deposition throughout the time-period of multi-time analysis, yielding a structural model for film growth having a single uniform bulk layer.

**Figure 5:**
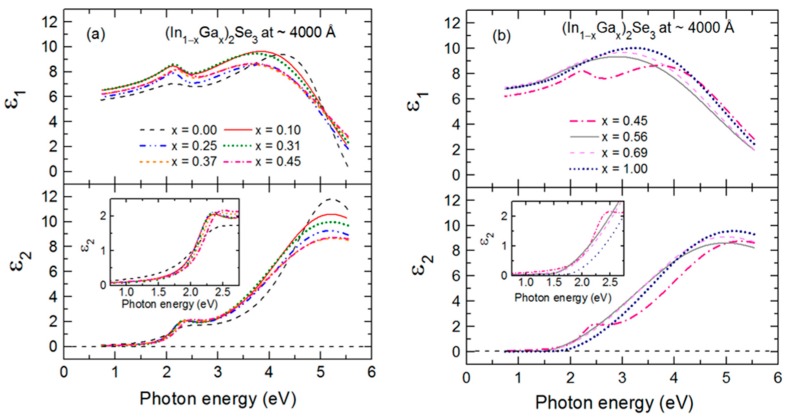
Parametric forms of the complex dielectric function spectra (ε_1_, ε_2_) for IGS films at bulk layer thicknesses within the range of 3500–4100 Å measured at 400 °C for (**a**) *x* ≤ 0.45 and (**b**) *x* ≥ 0.45. These (ε_1_, ε_2_) spectra generate the best fits to the results obtained by inversion at the center of the time range used for multi-time analysis. A high energy Tauc-Lorentz oscillator is used over the full range of *x*, whereas a bandgap CP oscillator is clearly observable in the (ε_1_, ε_2_) spectra only for the IGS depositions with *x* ≤ 0.45.

As an example of the fitting achieved continuously versus time in the multi-time analysis, RTSE data in ψ at the specific photon energies of 0.735, 1.620, 2.546 and 5.912 eV are plotted together with the best fit results in Figure 4 for the IGS deposition with *x* = 0.31. Deviations between the data and best fit in Figure 4 occur not only due to noise in the data (particularly at the lowest and highest photon energies of the range) but also due to limitations in the accuracy of the single bulk layer model and the b-spline dielectric function. Figure 5 shows the final parametric (ε_1_, ε_2_) spectra obtained by fitting the inversion results near the center at t* of the multi-time analysis ranges using the oscillator model, which includes single CP and TL oscillators. The resonance energy parameter of the CP oscillator represents the fundamental bandgap *E_g_*_1_. In contrast, the bandgap of the TL oscillator described as a Tauc gap *E_g_*_2_ serves as a low energy absorption onset for a higher energy Lorentz oscillator with a resonance energy of *E*_02_. The CP and TL oscillators are also defined by their individual amplitudes (*A*_1_, *A*_2_) and their broadening values (Γ_1_, Γ_2_), respectively. The phase and exponent of the bandgap CP oscillator are fixed at 0° and 0.5, respectively, and the constant contribution to the ε_1_ spectra is fixed at unity. 

**Figure 6:**
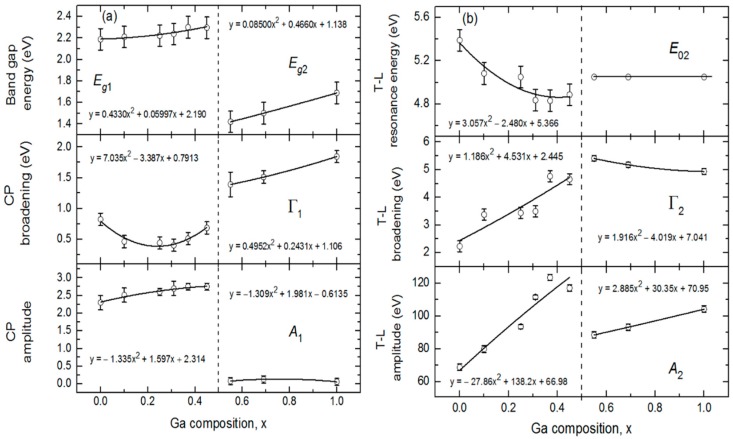
(**a**) (Top) Critical point (CP) resonance energy *E_g_*_1_ (*x* ≤ 0.45) or Tauc-Lorentz (TL) oscillator bandgap *E_g_*_2_ (*x* ≥ 0.56) as a function of the [Ga]/{[In] + [Ga]} atomic ratio (*x*) for IGS; (center) CP broadening parameter Γ_1_ and (bottom) amplitude *A*_1_ as functions of *x*. (**b**) (Top) The TL oscillator resonance energy (*E*_02_), (center) broadening parameter Γ_2_ and (bottom) amplitude *A*_2_ all plotted as functions of *x*. The TL oscillator resonance energy *E*_02_ has been fixed to the value of 5.05 eV for Ga compositions *x* > 0.56 whereas it is allowed to vary for *x* ≤ 0.45. The constant contribution to the real part of the complex dielectric function ε_1,∞_ is fixed at unity throughout. These results are appropriate for an IGS temperature of 400 °C and a bulk layer thickness in the 3500–4100 Å range.

Figure 6 shows the variations of the bandgaps, resonance energies, amplitudes and broadening parameters of the CP and TL oscillators versus alloy composition *x* over the full range of *x*. The In- and Ga-rich sides of IGS series have different oscillator characteristics and trends with composition as reflected in Figure 6. For *x* ≤ 0.45, the bandgap CP oscillator is clearly visible in (ε_1_, ε_2_) at *E_g_*_1_ ~ 2.2–2.3 eV and exhibits a wide minimum in the broadening parameter Г_1_ centered in the range *x* = 0.20–0.30. For *x* ≤ 0.45, the Tauc gap associated with the high energy TL oscillator is fixed at the bandgap CP energy in order to prevent absorption associated with the TL oscillator from appearing below the bandgap CP energy. In contrast to the behavior for *x* ≤ 0.45, the amplitude of the CP oscillator is essentially zero for *x* ≥ 0.56, meaning that the signature of the fundamental bandgap disappears from the optical response for the Ga-rich alloys. In its place, a very gradual low energy absorption onset is observed that can be simulated with the low energy tail of the TL oscillator with a Tauc gap that now controls the absorption onset due to the absence of the CP oscillator. This gap *E_g_*_2_ increases from 1.42 to 1.69 eV as *x* increases from 0.56 to 1.00. In the final analysis for *x* ≥ 0.56, the Lorentz oscillator resonance energy associated with the TL is fixed at *E*_02_ = 5.05 eV, an average obtained when all TL parameters are varied for the three samples. This was done to stabilize the fit and obtain a systematic variation in *x* for the other TL parameters for *x* ≥ 0.56. 

Expressions describing the dependence of each variable oscillator parameter on *x* for *x* ≤ 0.45 and *x* ≥ 0.56 were obtained by fitting the results for each parameter to a polynomial function of *x* as shown by the solid lines in Figure 6. These best fit polynomial functions are collected in Table 2 and Table 3. The parametric expressions of greatest interest for CIGS solar cells are those relevant to Ga contents with *x* ≤ 0.45 in Figure 6 and Table 2. These expressions span the composition range *x* = 0.20–0.40 where the most efficient solar cells are obtained [5,7,8,9,10]. Noteworthy trends in Figure 6 that are evident from the polynomial fits with increasing *x* for *x* ≤ 0.45 include (i) a monotonically increasing bandgap CP from 2.19 eV at *x* = 0.00 to 2.33 eV at *x* = 0.45, (ii) a broad minimum in the CP width centered near *x* = 0.20–0.30, (iii) an increasing CP oscillator amplitude that tends to saturate for the depositions at the higher values of *x* and (iv) a decreasing resonance energy and an increasing broadening parameter and amplitude associated with the high energy TL oscillator. Additional trends occur in Figure 6 based on consideration of the full range of composition. First, it should be noted, however, that the amplitude of the CP observed clearly for *x* ≤ 0.45 is very small for *x* ≥ 0.56, nearly approaching zero. As a result, the increase in CP broadening with increasing Ga content for *x* ≥ 0.56 is not a meaningful feature in the interpretation. In contrast, the amplitude of the broad TL oscillator is quite strong for all compositions, and so the increases in the amplitude and bandgap associated with this oscillator and the reduction in its broadening parameter with increasing Ga content for *x* ≥ 0.56 in Figure 6 are obviously meaningful. Although the bandgap feature due to the CP is sharpest for *x* = 0.20–0.30, the smallest broadening parameters of the high energy oscillator over the two respective ranges of *x* in Figure 6b occur at *x* = 0.00 and *x* = 1.00.

It is of interest to compare the bandgaps for In_2_Se_3_ of 2.19 eV and Ga_2_Se_3_ of 1.69 eV from the parameters of Table 2 and Table 3 with available results in the literature. From the temperature dependence of the free exciton photoluminescence feature, the room-temperature bandgap of γ-phase In_2_Se_3_ films has been estimated as ~1.95 eV [43], whereas the bandgap of zinc blende Ga_2_Se_3_ is estimated from absorption measurements as ~2.0 eV [44]. The wider bandgap of 2.19 eV in Table 2 for the In_2_Se_3_ at 400 °C may be attributed to compositional differences and the narrower bandgap of 1.68 eV in Table 3 for Ga_2_Se_3_ also at 400 °C may have components due to the red shift with temperature and to the defects and disorder of the Ga-rich alloys prepared in this study. 

The differences in behavior in Figure 6a,b over the different ranges of *x* are consistent with the SEM and XRD results of Figure 1 demonstrating that for IGS with *x* = 0.00 and 0.31, a large grained structure is found whereas for IGS with *x* = 0.56 and 1.00, the grains are much smaller. Evidently, it is the observed transition in grain structure between *x* = 0.31 and 0.56, rather than a transition in crystallography, that accounts for the relatively abrupt transition from a well-defined direct bandgap with a strong CP amplitude to a gradual absorption onset with a negligible CP amplitude. Although a gradual absorption onset could be characteristic of an indirect bandgap semiconductor, in this case it is more likely to arise from a defective or nanocrystalline film structure [45]. Additional observations of the SEM images of selected IGS films as shown in Figure 1 reveal behavior that supports the interpretation of the results in Figure 6a, in particular the broadening parameter Г_1_ associated with the bandgap CP. The reduced Г_1_ value between *x* = 0.00 and *x* = 0.31 suggests a longer excited state lifetime and a longer excited carrier mean free path. This in turn indicates a larger grain size if grain boundary scattering limits the mean free path, or a lower defect density if defect scattering is the limiting mechanism. Thus, the reduction in Г_1_ in Figure 6a is consistent with the increase in grain size observed from the SEM images in Figure 1 for an increase in IGS composition from *x* = 0.00 to *x* = 0.31. 

In contrast, the width of the high-energy feature Г_2_ represented by the TL oscillator may be controlled by broadening due to alloying over the two composition ranges, with Ga alloying from *x* = 0.00 to 0.45 and with In alloying from *x* = 1.00 to 0.56. Alternatively, if the TL oscillator serves to simulate multiple transitions, such parameter variations with *x* may be due to electronic structure variations. Neither effect would be evident in the SEM images and may explain why Г_2_ in Figure 6b increases with increasing *x* from *x* = 0.00 to 0.31 and increases as well with decreasing *x* from *x* = 1.00 to 0.56, even though the grain size from SEM increases with alloying from each endpoint. 

The most interesting feature of the trends in Figure 6a and Table 2 is the behavior in Г_1_, the broadening parameter of the bandgap CP. In fact, the composition range of the minimum in this parameter is close to that yielding the highest efficiency CIGS solar cells. It has been proposed that the origin of the optimum CIGS solar cell efficiency near *x* ~ 0.20–0.30 arises from a minimum in the concentration of volume defects due to crystallographic disorder for the ideal lattice parameter ratio of *c/a* = 2 near this composition [10,46]. The results for the SEM of Figure 1 and in particular for Г_1_ of Figure 6a, lead to the interesting possibility that the optimum three-stage CIGS at *x* ~ 0.20–0.30 is critically linked to the optimum IGS structural properties such as the largest grain size and/or lowest grain boundary related defect concentration associated with the IGS from which CIGS is formed via Cu diffusion in stage II. 

For useful applications in IGS deposition monitoring, the IGS (ε_1_, ε_2_) spectra can be summarized in the form of a database of coefficients. These coefficients generate polynomials in the Ga composition *x* in Table 2 and Table 3 that in turn describe the parameters to be used in the analytical expressions for the (ε_1_, ε_2_) spectra. It should be recalled that because RTSE is performed at the IGS deposition temperature, the expressions generated by Table 2 and Table 3 are relevant to describe the (ε_1_, ε_2_) spectra only near 400 °C. Room temperature (ε_1_, ε_2_) spectra are not useful for this optical property database since the IGS films prepared in stage I of the three stage co-evaporation process are not cooled to room temperature after deposition. Instead, they are heated to the stage II temperature, which is typically in the range of 540 to 620 °C, depending on the softening temperature of the SLG used as a substrate. Because all parameters in the analytical expressions for the (ε_1_, ε_2_) spectra of IGS at 400 °C are expressed in terms of a single parameter, the composition *x*, the (ε_1_, ε_2_) spectra can be generated for any given composition over these two individual ranges in *x*. This capability can be used in conjunction with least squares regression, employing the IGS composition as a free parameter along with the thicknesses d_b_ and d_s_ in RTSE analysis. 

**Figure 7:**
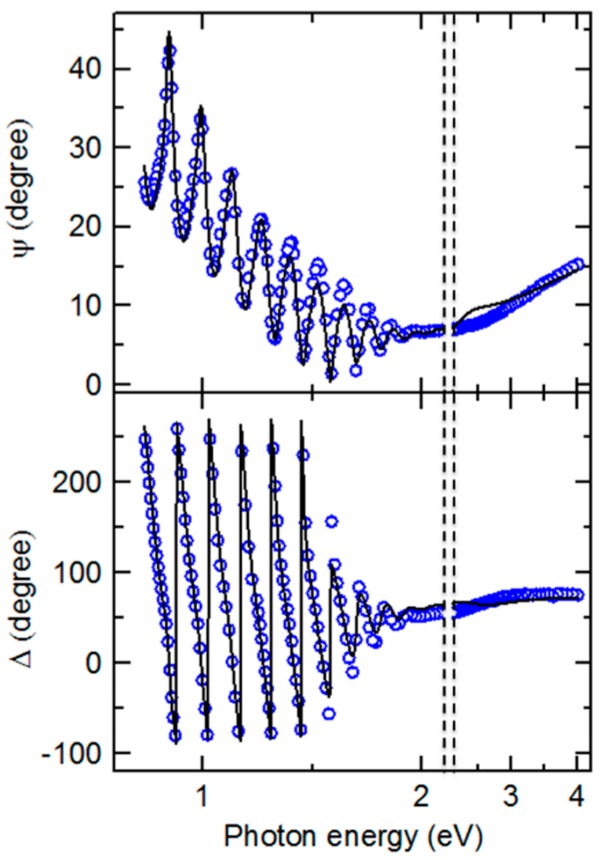
Ellipsometric spectra (points) obtained in-situ at the end of an IGS deposition at a substrate temperature of 400 °C before initiation of the second stage of CIGS deposition. Also shown is the best fit (solid lines) using the structural model and best fit parameters of Figure 8. Different linear scales are used over the ranges of 0.74–2.1 eV and 2.1–4.0 eV for greater clarity of the fringe pattern.

**Figure 8:**
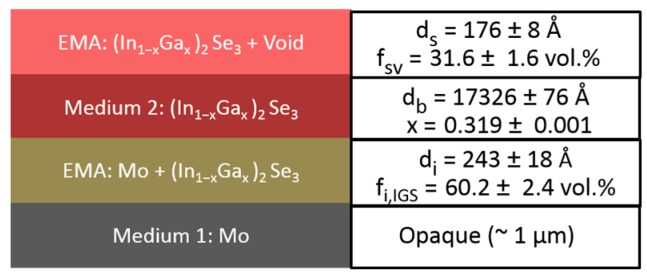
Schematic of the structural model with best fit parameters and confidence limits obtained in the analysis of the data in Figure 7. The parametric expression for the (ε_1_, ε_2_) spectra of IGS at 400 °C defined in Table 2 is used for the determination of the IGS composition. The roughness and interface contents f_sv_ and f_i,IGS_ represent the void and IGS component of the EMA layers, respectively.

The results of such an IGS compositional analysis using the parametric expressions of Figure 6 and Table 2 are shown in Figure 7 and Figure 8. This analysis was applied to a single pair of (ψ, Δ) spectra collected by in-situ SE at the end of the deposition of an IGS layer at 400 °C on the surface of a thicker (~1.0 μm) Mo bilayer as the stage I fabrication step of a three-stage CIGS solar cell. Figure 7 shows the SE data in the form of (ψ, Δ) along with the best fit using the six-parameter model of Figure 8, which is based on the schematic structure of Figure 3. The parameters include the interface, bulk and surface roughness layer thicknesses, the IGS and void contents in the interface and surface roughness layers, respectively, and the IGS composition *x*, which defines the (ε_1_, ε_2_) spectra of the IGS component of all three layers. Figure 7 shows that the best fit is poor at the high photon energies, possibly a consequence of applying the (ε_1_, ε_2_) database for 4000 Å IGS to model 1.7 μm IGS. The thicker surface roughness layer on the 1.7 μm IGS may also play a role in the poor fit at high photon energies. In spite of this, the interference fringe patterns in the data at low energies and in particular their damping behavior near the IGS absorption onset are reproduced quite accurately. Aside from the limitations of the best fit, some positive indicators are observed. First, the deduced Mo/IGS interface layer thickness of 243 Å and the IGS content within the interface layer of 60.2 vol % are very close to the surface roughness thickness and void content, respectively, deduced for the underlying Mo before IGS deposition. This suggests that the interface layer derives from the roughness on the Mo and that the voids in this roughness layer are completely filled with IGS in the initial time period of IGS deposition. In addition, the deduced composition *x* is within ~0.02 of the intended value of *x* = 0.30 for the solar cell and within ~0.01 of the depth averaged value of 0.31, obtained from ex-situ SE of the final solar cell assuming a composition profile consisting of two linear segments [47].

The results of Figure 7 and Figure 8 demonstrate that the IGS composition can be determined from in-situ and real time SE measurements. Once the parametric form for the IGS (ε_1_, ε_2_) versus *x* is available, along with the Mo dielectric function, analysis results such as those in Figure 8 can be obtained in analysis times on the order of seconds. Thus, if the deposition chamber is fitted with RTSE instrumentation, *x* can be determined from measurements during as well as after stage I deposition without removing the sample from the chamber and without even interrupting the deposition process. It should be emphasized, however, that the application of the IGS (ε_1_, ε_2_) database in Figure 6 and Table 2 is restricted to deposition temperatures near 400 °C. A key feature of the IGS (ε_1_, ε_2_) spectra that provides compositional sensitivity is the shift in the CP to high energy with increasing *x* evident in the inset of Figure 5. Although the shift is relatively weak, ~0.14 eV for 0 ≤ *x* ≤ 0.45, in the case of a thick film, the CP characteristics control the rapid damping of the interference fringes evident in the transition from the oscillatory pattern in (ψ, Δ) to the smoothly varying spectra with increasing photon energy in Figure 7. Furthermore, the results of Figure 7 and Figure 8 would suggest that the (ε_1_, ε_2_) database in Table 2 from the ~3500–4100 Å IGS thickness range is quite robust as it has provided IGS composition even for a thick IGS layer obtained at the end of deposition on a solar cell relevant substrate with a thick Mo back contact layer leading to significant roughness at the Mo/IGS interface. One must further explore the limitations of the Table 2 database in studies of the effects of thickness on the IGS complex dielectric function. The results of such studies will be described in detail in the next section.

### 3.2. Evolution of the Complex Dielectric Function with IGS Thickness

The central time for the range of multi-time analysis described in Section 3.1 was selected as that corresponding to a bulk layer thickness of 3500–4100 Å. This thickness is approaching the mid-way point of the 9000 Å deposition for the series of films prepared with different compositions on Si/SiO_2_/Mo substrates. This thickness was also selected to achieve a balance between the highest accuracy and broadest relevance for the parametric expressions of Figure 6, Table 2 and Table 3. As will be described later in this section, for thinner films, the (ε_1_, ε_2_) spectra are not representative of the thicker films used in devices, possibly due to a smaller grain size or a much higher defect density than the thicker films. For thicker films, however, one must be concerned with the influence of depth non-uniformities on the RTSE analysis results; the effects of these become more pronounced with increasing thickness. In addition, the surface roughness on the IGS increases with increasing thickness, which leads to greater challenges in extracting accurate (ε_1_, ε_2_) spectra of the bulk layer beneath the surface roughness layer.

For the key IGS layer with *x* = 0.30 deposited on SLG coated with an ~8000 Å Mo layer, the structural evolution and (ε_1_, ε_2_) spectra were obtained by performing a number of multi-time analyses over ranges centered at different times. In these analyses, the three-layer optical model is applied as shown in Figure 3. In addition, the Mo/IGS interface layer was fixed at the value of 164 Å, as deduced from a measurement of the Mo surface roughness thickness at room temperature prior to substrate heating and deposition. It is also assumed that the voids in the 38/62 vol % Mo/void mixture deduced to characterize the Mo surface roughness layer are completely filled by IGS during interface formation as will be described in Section 3.3. It should be noted that if the (ε_1_, ε_2_) spectra evolve with thickness, leaving an optical property depth profile built into the film, then the three-layer model in Figure 3 is an approximation since it assumes a bulk layer having uniform (ε_1_, ε_2_) spectra with depth. This approximation becomes closer to reality with increasing photon energy above the bandgap since a small absorption depth implies that the light does not probe the full thickness range of the non-uniformity. Near the bandgap, however, one must be concerned with possible distortions of the deduced (ε_1_, ε_2_) spectra as a result of neglecting the depth non-uniformity. 

In an initial multi-time analysis for each selected thickness range, a Kramers-Kronig consistent b-spline model for the (ε_1_, ε_2_) spectra is assumed for the bulk layer as described in Section 3.1. From this analysis, the time evolution of the bulk and surface roughness layer thicknesses is determined. A summary of the analysis details and thickness results are presented in Table 4 for the IGS deposition with *x* = 0.30, listed in order of the initial time t_i_ of the multi-time range given in the first column. In addition to t_i_, the final time t_f_ and the center time t* are given, as well as the best fit bulk layer thicknesses d_b_ at t_i_, t_f_ and t* along with the surface roughness thickness d_s_ and its void content f_v_ at t*. For the surface roughness layer, a 50/50 vol % mixture of IGS/void is assumed for roughness layer thicknesses <90 Å, whereas the roughness layer composition is allowed to vary for thicker roughness layers. As described in Section 3.1, the Bruggeman effective medium approximation is used to determine the (ε_1_, ε_2_) spectra of these roughness layers using the spectra of the IGS/void components and the void fraction values as input. Also shown in the last column of Table 4 is the IGS deposition rate, given as the derivative of effective thickness d_eff_ versus time. The deposition rate in terms of d_eff_ should be constant over the deposition time if the fluxes from the In and Ga evaporation sources are stable. The results in Table 4 suggest that the rate is constant at 10.2 Å/s within ~±3% over the first 10 min of deposition. A ~7% higher rate than the average over the initial 10 min is determined at the end of deposition. This higher rate is outside the confidence limits of the measurement. Such fluctuations in rate during longer depositions due to variations in the source fluxes may lead to compositional non-uniformities in the IGS film.

**Figure 9:**
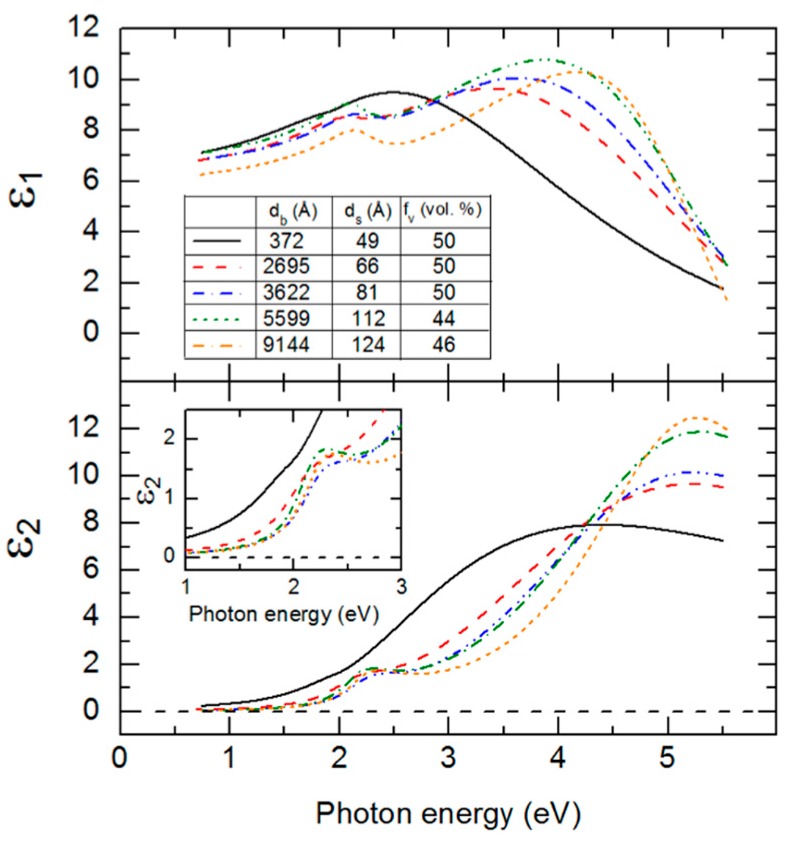
Plots of the analytical expressions for the (ε_1_, ε_2_) spectra of an IGS thin film with *x* = 0.30 obtained by fitting the spectra deduced by inversion at the centers of five different time ranges used in the multi-time analysis of RTSE data. A table is included in the upper panel that provides the bulk layer thickness, surface roughness layer thickness and void volume percentage in the roughness layer at the center time of each analysis range. The inset of the lower panel depicts the onset in ε_2_ near the fundamental bandgap, represented by the CP resonance energy. All (ε_1_, ε_2_) spectra depicted here are relevant for the substrate temperature of 400 °C.

The structural parameters of Table 4 including d_b_, d_s_ and f_v_ deduced at t* are used in an inversion procedure to extract the (ε_1_, ε_2_) spectra from the (ψ, Δ) spectra at t*. Such inversions at the five center times in Table 4 lead to a series of (ε_1_, ε_2_) spectra at different bulk layer thicknesses. Each set of (ε_1_, ε_2_) spectra obtained by inversion is fitted assuming the same parametric expression that was applied for the IGS composition series within the narrow 3500–4100 Å range of thickness. In addition to the expression, the constraints on the parameters in the analyses were the same as well. The best fit parametric forms of the (ε_1_, ε_2_) spectra of IGS for the different bulk layer thicknesses of Table 4 are presented together for comparison in Figure 9.

**Figure 10:**
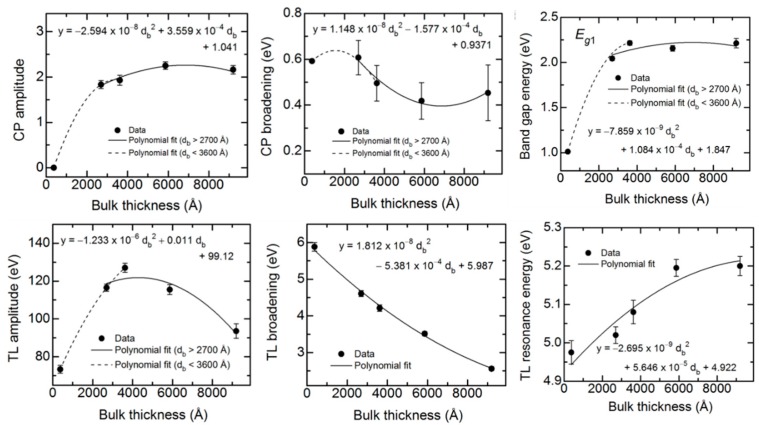
Best fit critical point (CP) and Tauc-Lorentz (TL) oscillator parameters (points) plotted versus bulk layer thickness for (In_1−x_Ga_x_)_2_Se_3_ with *x* = 0.30, along with best fit polynomial relationships (lines). For Г_2_ and *E*_02_, a single polynomial spans the full range of bulk layer thickness, whereas for all other parameters, two polynomial segments are needed. For the latter set of parameters, the equation provides the higher thickness polynomial segment. Over the low thickness range (broken lines), the (ε_1_, ε_2_) spectra show characteristics of a transition from a disordered semiconductor to a larger grain polycrystalline semiconductor as the bandgap CP amplitude increases above a small value. The best fit polynomial equations obtained in the fits to these data over the one or two thickness ranges are provided in Table 5. The CP phase and exponent are set at 0° and 0.5, respectively, and the constant contribution ε_1,∞_ is fixed at unity. The TL oscillator bandgap is fixed at the deduced CP resonance energy value shown at upper right. When the CP amplitude is small, however, the TL oscillator bandgap controls the absorption onset.

Among the ten parameters that could be varied, namely five for the CP oscillator, four for the TL oscillator and the constant ε_1∞_, only six are varied. The CP phase and exponent are fixed at 0° and 0.5, respectively and ε_1,∞_ is fixed at unity. In addition, the TL bandgap energy is equated to the CP resonance energy. The best fit variable parameters in the analytical expression for these (ε_1_, ε_2_) spectra are plotted versus d_b_ at t* in Figure 10. In order to quantify the observed thickness effects, polynomial functions describing the dependence of each variable oscillator parameter on bulk layer thickness were obtained in as many as two segments. The resulting polynomial functions associated with each parameter, shown by the lines in Figure 10, are summarized together in Table 5.

As is clear from the results in Figure 9, the (ε_1_, ε_2_) spectra at an IGS bulk layer thickness of 372 Å are significantly different from the spectra at later times. The thin layer (ε_1_, ε_2_) spectra exhibit only the high energy TL oscillator with no evidence of the lower energy CP oscillator, the latter identifying the crystalline phase bandgap. Because of the abruptness of the Mo/IGS interface indicated by the EDS profile in Figure 2, the different behavior of the (ε_1_, ε_2_) spectra at 372 Å is not attributed to a different initial growth material such as MoSe_2_ that could be generated by reaction of the initial deposition flux with the Mo surface. The lower temperature of the stage I process may account for the proposed absence of a reaction as indicated previously [36]. Instead, the thin layer material appears to be highly defective or disordered IGS with a TL oscillator bandgap of ~1 eV, which is much lower than the CP energy of the IGS determined at the later times. As a result of the two different characteristics of the (ε_1_, ε_2_) spectra, the polynomials in Figure 10 and Table 5 are given over the two overlapping thickness ranges of 370–3600 Å and 2700–9100 Å, indicated by the broken and solid lines, respectively, in Figure 10. For the range of smaller thickness values, it is clear that the database for the (ε_1_, ε_2_) spectra in Figure 5 and Table 2 obtained over the 3600–4100 Å range cannot be used. More detailed studies spanning the 370–3600 Å range are needed to provide an appropriate database that may describe the dependence of the (ε_1_, ε_2_) spectra on *x* and d_b_ when the CP is suppressed and a single TL oscillator may be used to fit the inversions. Further study of the structural evolution and (ε_1_, ε_2_) spectra over this range of thickness for the different IGS compositions will be presented in Section 3.3. 

For the 2700–9100 Å range of larger thicknesses where the bandgap CP is clearly observed, the associated CP parameter variations with thickness are relatively weak at least within the confidence limits, as indicated in Figure 10. This is also observed in the similarity of the absorption onsets given in the Figure 9 inset, particularly for thicknesses ≥3600 Å. In contrast, the TL oscillator parameters show larger variations over the 2700–9100 Å thickness range. The TL oscillator amplitude in Figure 10 decreases systematically with thickness above 3600 Å with a variation outside of the confidence limits. In contrast to expectations from Figure 10, the high energy TL oscillator is increasing in peak height with increasing thickness in the (ε_1_, ε_2_) spectra of Figure 9. This apparent inconsistency arises from the fact that the prefactor in the TL oscillator equation for ε_2_ incorporates not only the amplitude but also the broadening parameter and the resonance energy. As a result, the amplitude *A*_2_ more closely reflects the integral of the oscillator, rather than its peak height. The strong systematic decrease with thickness in the broadening parameter Г_2_ leads to a decrease in the TL oscillator integral in spite of the increase in peak height in Figure 9 and, thus, a decrease in the value of *A*_2_ in Figure 10. 

An apparent decrease in the broadening from thin (2700 Å) to thick (5900 Å) films is also observable for the CP, whereas at the largest thickness of 9100 Å the continuation of this trend is unclear. A decrease in the broadening parameter with thickness is a general indicator of a reduction in defect density or disorder, or an increase in grain size. It may be possible to couple the two broadening parameters and other CP and TL oscillator parameters as needed, to a single excited electron mean free path λ. In fact, this second parameter λ may serve along with *x* as a two-parameter description of the (ε_1_, ε_2_) spectra for both compositional and grain size analysis of IGS films by RTSE. Such a coupling of broadening parameters has been demonstrated for the multiple CPs in the (ε_1_, ε_2_) spectra of CdS and CdTe thin films [48]. In addition, a simultaneous change in void content f_bv_ may occur with increasing d_b_, and this could be added as a third parameter in addition to *x* and λ for the simulation of the (ε_1_, ε_2_) spectra of IGS films to account for possible density variations with thickness. These approaches are restricted to thicker films with d_b_ > 2700 Å where the same form of the complex dielectric function is obtained. 

A key observation of this section is the systematic thickness dependence in Figure 9 over the high energy range where the TL oscillator dominates, which then appears to suggest that the polynomials in Figure 6 and Table 2 are relevant strictly for thicknesses in the range of 3500–4100 Å. Variations in the ε_1_ spectra in Figure 9 also occur over the low photon energy range. In fact, the differences in the overall shapes of the (ε_1_, ε_2_) spectra for different thicknesses in Figure 9 motivate a need for adjusting the parameters of Table 2 and Table 3 if the parametric expressions are to be valid for IGS films with largely different thicknesses. On this basis, an explanation for the apparent robustness of the fitting as indicated in Figure 7 and Figure 8 for a 1.7 μm thick IGS layer must be provided. It is evident from the inset in Figure 9 that no variation in ε_2_ with thickness occurs for thicknesses ≥3600 Å when 0 < ε_2_ < 0.5, which is the case for photon energies 0.74 < *E* < 1.9 eV. It is this range that controls the variation in Figure 7 from undamped fringe pattern to full opacity with increasing photon energy. This conclusion is drawn from the fact that ε_2_ ~ 0.2 generates an absorption depth *d*_0_ ~ *n*λ/2πε_2_ ~ 1.7 μm, where λ is the wavelength and *n* is the index of refraction. In conclusion, an unchanging ε_2_ spectrum with thickness near the absorption onset in Figure 9 may help to explain the success of the analysis of Figure 7 and Figure 8. Finally, the thickness dependence in the ε_1_ spectra in Figure 9 over this same low photon energy range may be compensated by variations in the film thickness in the modeling of Figure 7 and Figure 8, again accounting for the robustness of the database of Section 3.1. A more advanced database including variations in parameters that account for the dependence on thickness, however, may lead to improved fits in the high energy range of Figure 7.

### 3.3. Early Stage Dielectric Function and Structural Evolution 

RTSE is sensitive to monolayer level nucleation, coalescence and growth processes through precise measurements of bulk and surface roughness layer thicknesses. It also provides high sensitivity to the (ε_1_, ε_2_) spectra of materials even in ultrathin (~10 Å) layers [38,49,50]. Through measurements of the (ε_1_, ε_2_) spectra, RTSE has the potential to distinguish small differences in the alloy compositions of deposited thin film materials and can detect small density deficits in the films, which affect (ε_1_, ε_2_) as well. Considering the results of Section 3.1 and Section 3.2, it is relevant to explore the sensitivity of the (ε_1_, ε_2_) spectra to IGS composition in the early stage of growth and thus the potential capability of real time SE for compositional analysis in this early stage.

**Figure 11:**
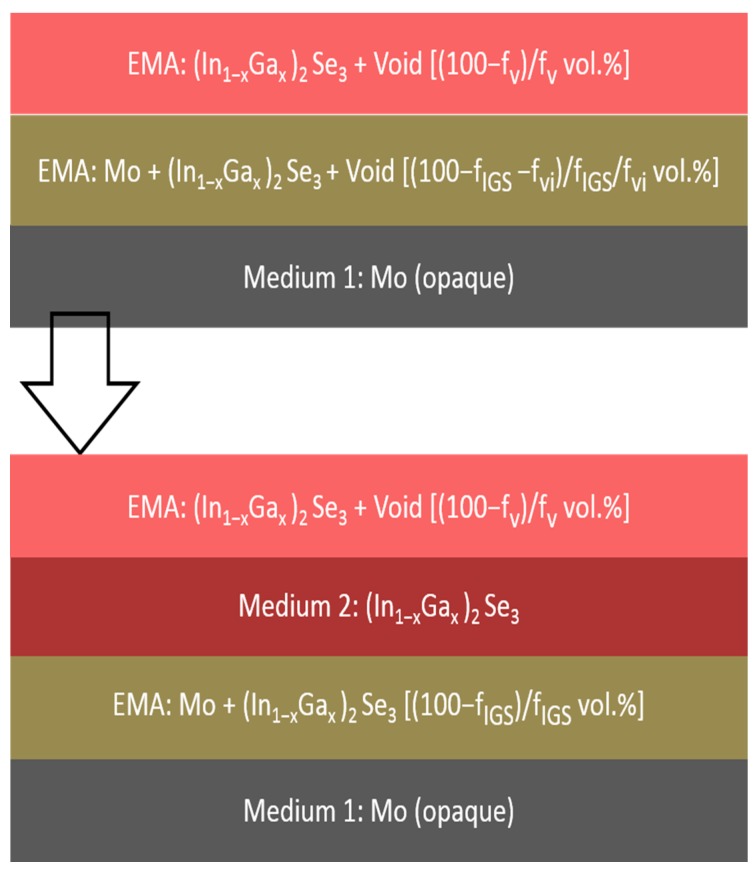
Structural models applied in two time regimes for analysis of RTSE data collected in the initial stage of IGS film growth on a rough Mo surface. In the first regime (top), the voids in the Mo surface roughness layer are filled by IGS as substrate-induced surface roughness associated with the IGS film develops. In this regime, no IGS bulk layer is observed and as a result, the regime is characterized by a two-layer model. The first regime transitions into the second regime (bottom) once the Mo surface roughness layer has been completely converted to a Mo/IGS interface roughness layer. In this second regime, the IGS bulk layer develops and increases in thickness with an IGS surface roughness layer on top, resulting in a three-layer model.

The multi-time and inversion approaches for RTSE data analysis have been adapted to describe the early stages of IGS film growth, focusing on the specific depositions using Mo-coated, thermally-oxidized Si wafer substrates as presented in Section 3.1. Distinct structural models depicted in the upper and lower panels in Figure 11 are used in two time regimes. In the first regime (upper panel), the voids in the Mo surface roughness layer are filled in by depositing IGS material as substrate-induced IGS surface roughness develops simultaneously. In this regime, no IGS bulk layer is observed and as a result, the regime is characterized by only two layers as shown in the upper panel of Figure 11. These two layers consist of (i) a three-component Mo/IGS interface roughness layer having a fixed thickness and Mo content, assumed equal to those of the roughness layer determined for the uncoated Mo surface, along with a variable IGS content f_IGS_, and (ii) an IGS surface roughness layer of thickness d_s_ with a variable void content f_v_. Here, the thickness and void content of the surface roughness layer and the IGS content in the interface roughness layer are determined individually at each time point in the analysis. In the second regime (lower panel of Figure 11), the Mo surface roughness layer has been completely converted to a Mo/IGS interface layer. The IGS bulk layer then develops and increases in thickness with a surface roughness layer on top consisting of an assumed 50/50 vol % mixture of IGS and void, resulting in a three-layer model. The three layers from the substrate to ambient medium consist of (i) a Mo/IGS interface roughness layer, having a fixed thickness and Mo content assumed equal to those of the Mo roughness layer, (ii) an IGS bulk layer of thickness d_b_ and (iii) an IGS surface roughness layer of thickness d_s_. In this regime, the bulk and roughness layer thicknesses are determined individually at each time point in the analysis. 

The complete multi-time analysis procedure has been performed on RTSE data acquired for samples of different IGS composition in studies of the nucleation and growth of the films, in addition to determination of the early stage (ε_1_, ε_2_) spectra. The data analysis applied in this subsection centers on bulk layer thicknesses in the range of 350–410 Å. The strategy is similar to those applied to the IGS films with different compositions and bulk layer thicknesses in the range of 3500–4100 Å as described in Section 3.1 and to a single film of composition *x* = 0.30 and different thicknesses as described in Section 3.2. Table 6 summarizes the selected initial time (t_i_), center time (t*) and final time (t_f_) in multi-time analyses performed to obtain the early stage structural evolution and (ε_1_, ε_2_) spectra for IGS depositions with different Ga contents. In these analyses, a Kramers-Kronig consistent b-spline model is used initially for the (ε_1_, ε_2_) spectra with a node spacing of 0.1 eV. The best fit bulk layer thicknesses at t_i_, t* and t_f_, as well as the surface roughness layer thickness and void volume percentage in the roughness layer at t*, are also summarized in Table 6. The mean square error for each of the best fit multi-time analyses is given in the second-to-last column of the table, with the deposition rate in terms of effective thickness d_eff_ in the last column. A comparison of the deposition rates in Table 1 and Table 6 suggests that a systematic increase in the rates up to 10% occurs from the early stage to the 3500–4100 Å thickness range for these IGS depositions that was not evident from the *x* = 0.30 deposition of Table 4. The average of these effective thickness rate increases is ~7%. 

**Figure 12:**
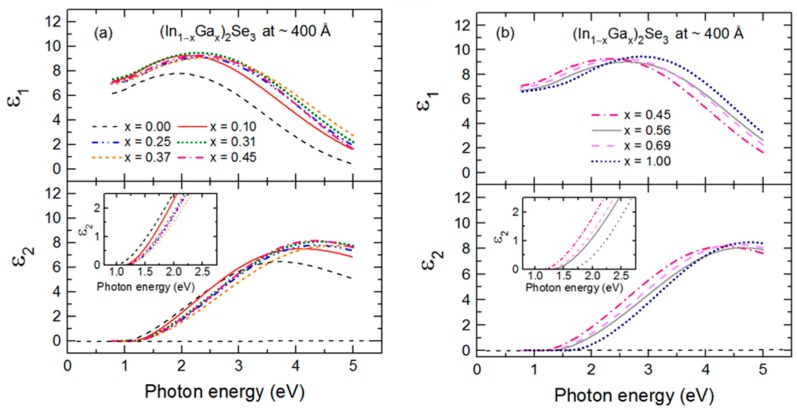
Parametric expressions for the complex dielectric functions (ε_1_, ε_2_) of ~350–410 Å thick IGS films measured at 400 °C for (**a**) *x* ≤ 0.45 and (**b**) *x* ≥ 0.45. The expressions incorporate a single Tauc-Lorentz oscillator with a high energy resonance for all the samples of different *x*. These (ε_1_, ε_2_) spectra are determined as the best fits of the results obtained by inversion at the center of the time range of the multi-time analysis.

The deduced structural parameters at t* in Table 6 enable exact inversion of the (ε_1_, ε_2_) spectra at this time, and these representations of the data are fitted using an analytical expression given by a single TL oscillator. An additional CP term in the expression, as required for larger bulk layer thicknesses and compositions *x* ≤ 0.45, does not provide a significant improvement in the fit for these analyses. Figure 12 presents the complete set of (ε_1_, ε_2_) spectra for IGS depositions of different *x*, relevant for a measurement temperature of 400 °C and for IGS bulk thicknesses near ~400 Å. The best fit parameters in the single TL model for the inverted (ε_1_, ε_2_) spectra are given in Table 7. Various weak trends appear in Table 7. The resonance energy *E*_02_ increases from 4.0 to 4.9 eV between *x* = 0 and *x* = 0.37, followed by a decrease and a saturation in the range from 4.6 to 4.8 eV at higher *x* values. The Tauc gap *E_g_*_2_ shows the minimum and maximum values of ~1.0 and 1.6 at *x* = 0 and 1, respectively, but with a rather weak variation for 0.1 ≤ *x* ≤ 0.45 with *x* values between 1.11 and 1.16 eV. The broadening parameter Г_2_ shows minimum values for *x* = 0.00 and 1.00, with an abrupt increase with *x* for 0.00 ≤ *x* ≤ 0.10 and a gradual decrease for *x* ≥ 0.56. Based on the trends in Table 7, one can conclude that (ε_1_, ε_2_) spectra obtained near ~400 Å do not exhibit a sufficiently sharp absorption onset to provide high compositional sensitivity in the early stage of IGS growth. Only after the CP oscillator associated with larger grain polycrystalline IGS emerges does compositional analysis become possible as demonstrated in Section 3.1. 

The initial nucleation and early stage growth behavior of the IGS can be characterized according to the structural evolution in Figure 11 by applying the best fit early stage (ε_1_, ε_2_) spectra in the two models. These (ε_1_, ε_2_) spectra are modeled as Kramers-Kronig consistent b-splines and deduced over the range of 350–410 Å. It is clear from the results for the composition *x* = 0.30, as studied in depth in Section 3.2, that the thin layer (ε_1_, ε_2_) spectra are needed, e.g., those at d_b_* = 408 Å as indicated in Table 6 for the analysis of the *x* = 0.31 composition, because they differ significantly from those obtained near 3600 Å. 

**Figure 13:**
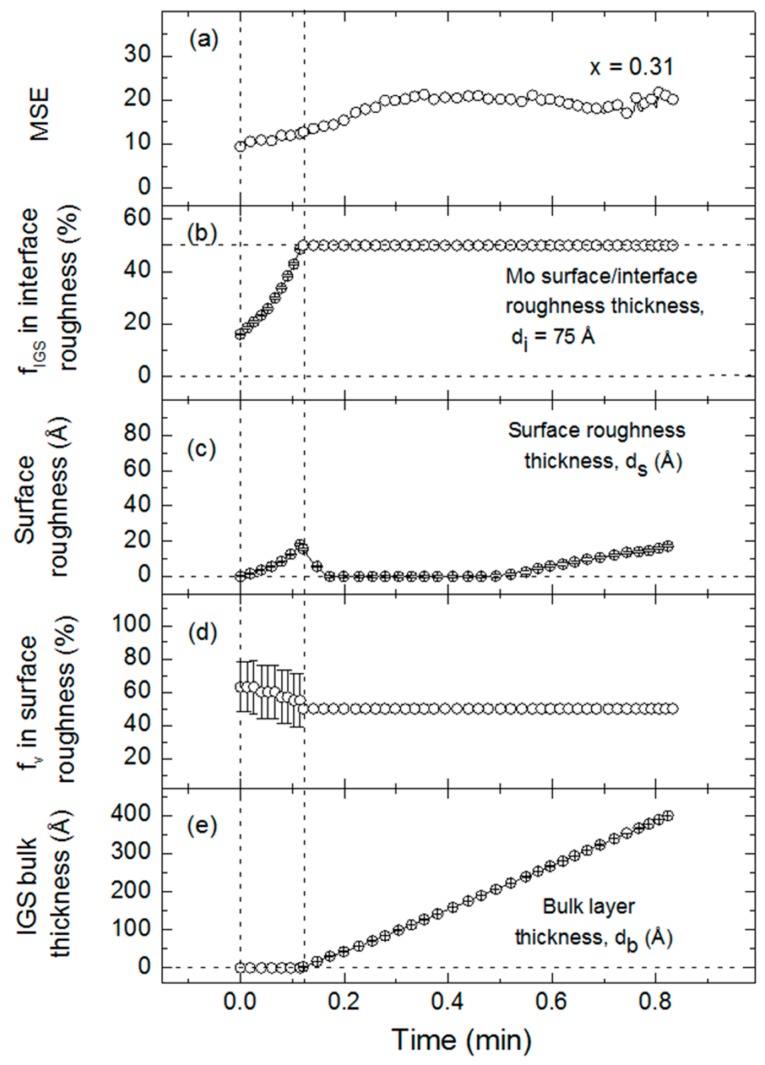
Best fit structural parameters and resulting mean square error (MSE) that characterize the first ~400 Å of (In_1−x_Ga_x_)_2_Se_3_ (*x* = 0.31) bulk layer growth on a Mo-coated, thermally-oxidized Si wafer substrate, including (**a**) the MSE describing the fit quality; (**b**) the IGS content in volume percent replacing the free space (assumed to be 50 vol % void at t = 0) in the modulations of the Mo surface roughness layer; (**c**) the IGS surface roughness layer thickness; (**d**) the void volume percent within the IGS surface roughness layer; and (**e**) the IGS bulk layer thickness. All results are plotted versus time after opening the shutter at t = 0 min to start the IGS deposition process which is designed to simulate stage I of three-stage CIGS co-evaporation.

Figure 13 shows the final results of this structural analysis for the *x* = 0.31 IGS film. The time evolution of the best fit MSE is presented in Figure 13a and the evolution of the best fit structural parameters according to the model of Figure 11 is presented in Figure 13b–e. The structural evolution panels depict the basic characteristics of interface filling, surface roughness development and bulk layer growth. Figure 13b shows the early stage evolution in which the assumed 50 vol % void content associated with the 75 Å thick roughness layer on the underlying Mo is replaced by IGS, leading to a rapid increase in the IGS content (f_IGS_) of the layer. Simultaneously, the IGS surface roughness layer thickness d_s_ increases and its void content f_v_ decreases as shown in Figure 13c,d, respectively, as the Mo surface roughness is conformally covered. After the void volume in the Mo surface roughness is completely filled by IGS to form a stable Mo/IGS interface roughness layer of composition 50/50 vol %, a bulk layer of thickness d_b_ can be incorporated into the model. In Figure 13e, the bulk layer is observed to follow expectations, increasing linearly with time from the onset of its growth. During initial bulk layer growth, the surface roughness on the IGS decreases rapidly in thickness as observed in Figure 13c, indicating that the substrate induced roughness is suppressed due to thin film structural coalescence. During bulk layer growth, Figure 13b,d are no longer interesting, as they simply show that the IGS content in the Mo/IGS interface roughness and the void content in the IGS surface roughness, respectively, are both fixed at 50 vol %. These assumptions are made for all analyses after the onset of bulk layer growth due to the complete filling of the Mo roughness and the relatively thin or in some cases negligible roughness layer during initial bulk layer growth. Interface filling, surface roughness development and a linearly increasing bulk layer thickness, as well as structural coalescence, are observed similarly for all other Ga compositions. 

**Figure 14:**
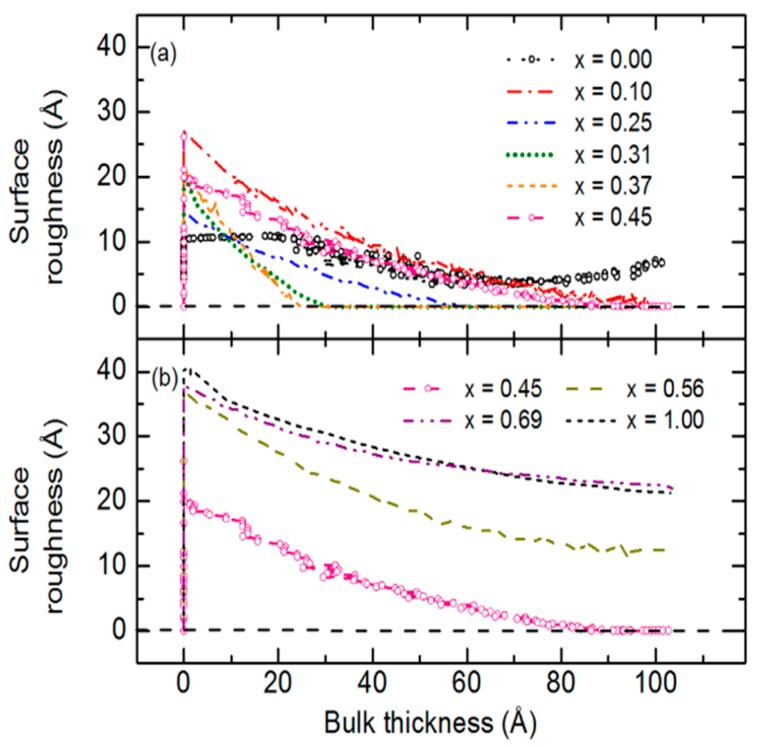
Evolution of the surface roughness layer thickness with bulk layer thickness for IGS films of different Ga compositions *x*; (**a**) 0.00 ≤ *x* ≤ 0.45 and (**b**) 0.45 ≤ *x* ≤ 1.00. The results focus on the first 100 Å of bulk layer thickness immediately after interface filling.

Next the focus of the discussion turns to the details of the IGS nucleation and growth process as depicted in Figure 13 for the composition *x* = 0.31 and in Figure 14 for all compositions. In the interface filling stage, IGS surface roughness development is interpreted as resulting from the IGS layer conformally covering the roughness on the Mo surface. Due to the presence of the relatively thick Mo roughness layer, in the range 69–88 Å, it cannot be determined whether interface filling occurs conformally layer-by-layer or as isolated nuclei that coalesce. In contrast, if the Mo was atomically smooth, any development of roughness in the initial stage could be attributed to a nucleation process. For the depositions of Figure 14, a roughness layer of thickness ranging from ~10 Å for In_2_Se_3_ to 40 Å for Ga_2_Se_3_ forms at the onset of bulk layer growth. Thus, for all depositions in Figure 14, the IGS roughness values do not approach those typical of the underlying Mo. This indicates that in the interface formation process, the evolving IGS surface smoothens in the transition from uncoated to completely coated Mo, and for all IGS depositions with *x* > 0.00, either the smoothening process or an atomically smooth surface is observed at least until a bulk layer thickness of 100 Å has been reached. 

Although non-monotonic behavior versus composition occurs in Figure 14, some observations appear conclusive. The minimum and maximum IGS surface roughness thicknesses of 10 Å and 40 Å at the onset of bulk layer growth occur for In_2_Se_3_ and Ga_2_Se_3_, respectively. The weakest smoothening effects subsequent to these onsets are observed for the In_2_Se_3_ deposition and for the three depositions with Ga compositions *x* ≥ 0.56. In fact, for In_2_Se_3_, roughening occurs after ~70 Å of bulk layer growth in Figure 14 whereas for Ga_2_Se_3_, weak smoothening continues over the 70–100 Å range of bulk layer growth. For IGS with 0.10 ≤ *x* ≤ 0.45, surface smoothening after the onset of bulk layer growth is not only rapid but also sufficient to generate atomically smooth surfaces. It is also interesting to note that for the samples with *x* = 0.31 and 0.37, the coalescence process occurs most rapidly, yielding a film with an atomically smooth surface after ~30 Å of bulk layer deposition. These same films also show longest time-period of atomically smooth surfaces. The behavior for all IGS depositions in Figure 14 is consistent with the single TL nature of the complex dielectric function of these films determined at bulk layer thicknesses of 350–410 Å in Figure 12. Consistency is suggested because highly disordered or even liquid films are expected to exhibit a single TL and can form atomically smooth surfaces upon coalescence [51]. A liquid film is a possibility due to the reduction in the melting point from the bulk that can occur in clusters and very thin films, as observed in RTSE studies of Ag thin film deposition [52]. It is of interest to note that the compositions with the most rapid smoothening effect during coalescence are those that exhibit the highest performance when incorporated into devices.

### 3.4. Intermediate and Later Stage Structural Evolution

In this section, the discussion will focus on the intermediate and later stage surface roughness evolution for the IGS thin films of different compositions. For the nine samples of the composition series, these stages are defined as spanning the thickness ranges over which the (ε_1_, ε_2_) spectra deduced at 3500–4100 Å and at the endpoint near 9000 Å, respectively, are relevant [53]. The results for the two stages can be compared with the roughness evolution associated with initial growth, which uses the (ε_1_, ε_2_) spectra deduced at thicknesses within the range of 350–410 Å. For the five different thickness ranges of the thicker IGS deposition with *x* = 0.30, the (ε_1_, ε_2_) spectra obtained by multi-time analysis using a b-spline model were applied, as described in Section 3.2. Although the complex dielectric function is varying with thickness throughout the deposition, the results for the surface roughness evolution presented in this subsection show clear trends with composition that support the validity of the overall analyses. 

**Figure 15:**
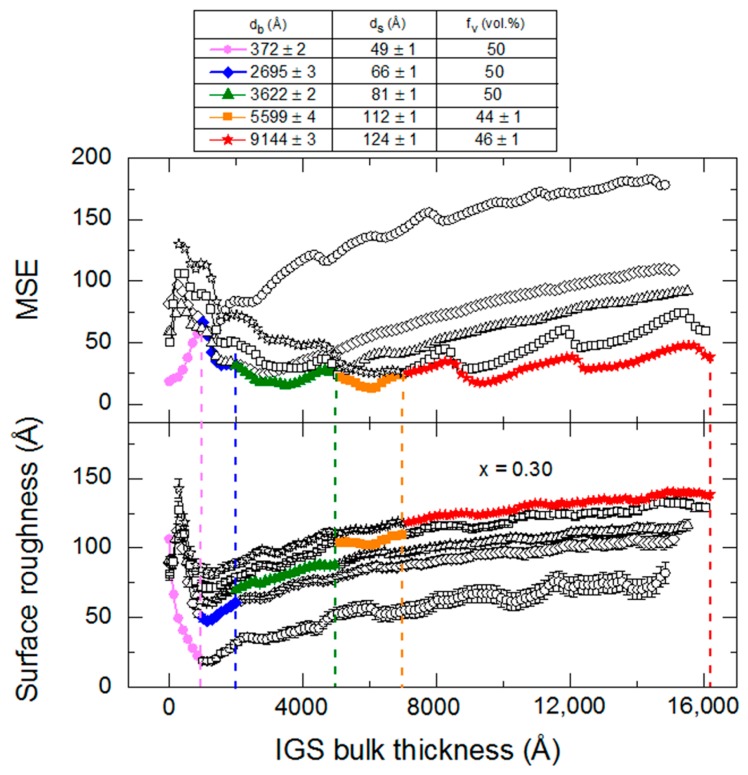
(**Lower** panel) Surface roughness evolution for the IGS film with Ga composition *x* = 0.30, obtained applying five different pairs of (ε_1_, ε_2_) spectra in turn deduced by multi-time analyses of RTSE data centered at five different bulk layer thicknesses; (**upper** panel) evolution of the mean square error (MSE) from the structural analyses using the five different (ε_1_, ε_2_) spectra. Solid points in the two panels highlight the lowest MSE results from among the five analyses. The inset above the panels shows the corresponding bulk layer thicknesses, surface roughness layer thicknesses and surface roughness layer void volume percentages at the center times of the five multi-time analyses.

In order to evaluate the effect of the thickness dependent (ε_1_, ε_2_) spectra on the deduced surface roughness evolution for an IGS film, the film with *x* = 0.30 from the thickness series of Section 3.2 was studied using five different pairs of (ε_1_, ε_2_) spectra in the structural analyses. For this film, the time evolution of the surface roughness and MSE, as determined using the five sets of (ε_1_, ε_2_) spectra, are shown in the lower and upper panels of Figure 15, respectively. A table of the bulk and surface roughness layer thicknesses and the surface roughness void contents at the center times of the multi-time analyses are reproduced from Table 4 above the panels in Figure 15. These structural parameters are obtained simultaneously with the five sets of (ε_1_, ε_2_) spectra modeled as b-splines. Considering the main part of Figure 15, clear offsets in the resulting values of surface roughness can be seen (lower panel), depending on the (ε_1_, ε_2_) spectra selected for analysis; however, the roughening trends are consistent among the five data sets. On the basis of the MSE (upper panel), the (ε_1_, ε_2_) spectra associated with the bulk thickness of 372 Å gives the lowest MSE from the onset of bulk layer growth to a thickness of only ~1000 Å. Because these (ε_1_, ε_2_) spectra are significantly different from those obtained in the analyses centered at 2695 and 3622 Å, as shown in Figure 9, the MSE obtained using the (ε_1_, ε_2_) spectra from the 372 Å center thickness (open circles) rapidly increases over this thickness range, and the resulting roughness evolution for IGS thicknesses above 1000 Å should not be considered accurate in this analysis. For the results using the (ε_1_, ε_2_) spectra from the four larger center thicknesses, the MSE increases are not as large. Selecting the lowest MSE results from among the five possible analysis results, the best fitting roughness evolution is expected to vary from ~110 Å at the lower thickness [results obtained with the (ε_1_, ε_2_) spectra deduced at the lowest thickness] to ~140 Å at the highest thickness [results obtained with the (ε_1_, ε_2_) spectra deduced at the endpoint]. The lowest MSE results are highlighted in the lower and upper panels of Figure 15 as the filled points. The larger roughness layer thicknesses in the early stage of growth for this *x* = 0.30 deposition compared with those for *x* = 0.31 in Figure 14 is substrate induced, i.e., caused by the much larger roughness on the surface of the thicker Mo film deposited as a solar cell back contact on SLG.

**Figure 16:**
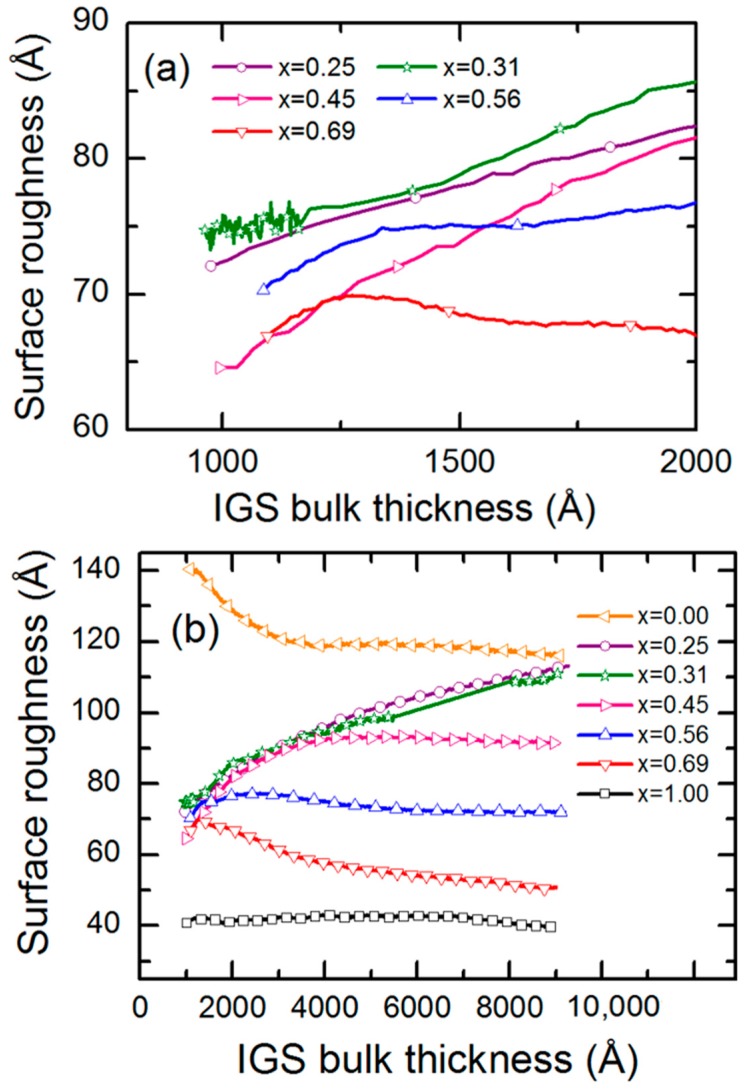
(**a**) Evolution of the surface roughness thickness for IGS films of selected composition *x* = [Ga]/{[In] + [Ga]} over the bulk layer thickness range of 1000–2000 Å; (**b**) extension of the results of (**a**) to a larger sample set and covering a wider range of bulk layer thickness. In both parts the (ε_1_, ε_2_) spectra used in the analysis were obtained from multi-time analysis at a center time within the range of 3500–4100 Å.

Figure 15 suggests that in a comparison of results for ~9000 Å thick IGS depositions of different compositions from Section 3.1, a reasonable approximation to the surface roughness evolution can be obtained over the bulk layer thickness range from 1000 Å to the end of the deposition using the (ε_1_, ε_2_) spectra deduced from multi-time analysis centered at 3500–4100 Å. Figure 16a shows these results for Ga compositions *x* = [Ga]/{[In] + [Ga]} selected for clarity over the bulk layer thickness range from 1000 to 2000 Å, and Figure 16b shows such results for a larger sample set and covering a wider range of bulk layer thickness to the deposition endpoint. For *x* ≤ 0.45, continuous roughening from 1000 to 2000 Å in Figure 16a suggests continuous crystallite evolution which generates growing protrusions at the surface, whereas for *x* = 0.69, a smoothening effect is observed, yielding a flatter surface characteristic of the stabilization of a fine-grained structure [53,54]. During the later stage of growth in Figure 16b, only the films with *x* = 0.25 and 0.31 show continued roughening throughout the growth process. All other films show either stabilized or smoothening surfaces at the end of deposition. 

**Figure 17:**
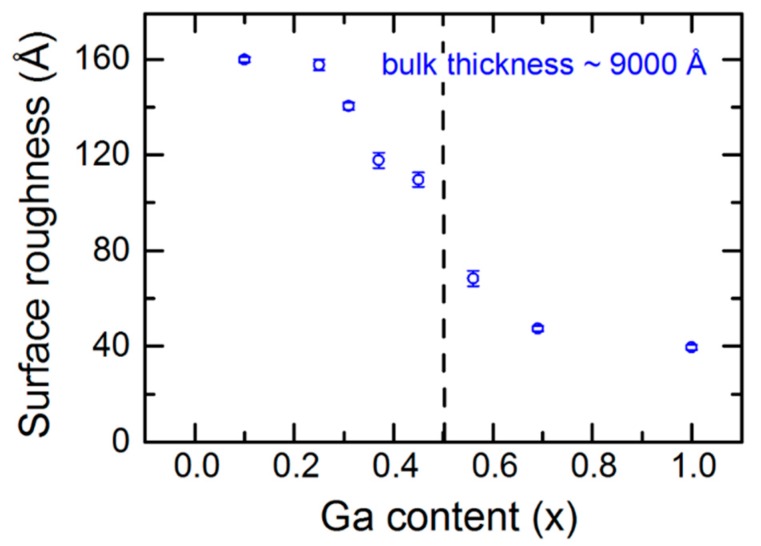
Variation in the IGS surface roughness layer thickness with Ga content *x* = [Ga]/{[In] + [Ga]} at the endpoint of each deposition near the bulk layer thickness d_b_ ~ 9000 Å. These surface roughness thickness values are obtained by using the complex dielectric functions of IGS at the end point thicknesses near 9000 Å.

The multi-time approach as described in Section 3.1 was also used for the determination of (ε_1_, ε_2_) spectra at the endpoint thickness near 9000 Å for the IGS depositions of the composition series of Section 3.1. The endpoint surface roughness layer thicknesses deduced from these (ε_1_, ε_2_) spectra are presented in Figure 17. Although these results exhibit a clear monotonic trend with composition, they also tend to show different magnitudes over the two ranges of composition *x* ≤ 0.45 and *x* ≥ 0.56. In fact, the surface roughness thickness shows a more rapid decrease when the *x* value increases above *x* ~ 0.45, which is likely to suggest a rapid reduction in the grain size, leading to smaller, stable protrusions above the surface. It is of interest to consider the results in Figure 17, in view of the (ε_1_, ε_2_) spectra versus *x* in Figure 5 and also in view of the SEM images and XRD patterns of the IGS films in Figure 1, obtained for the same set of IGS films. The rapid change in structure above *x* = 0.45 in Figure 17 is consistent with the loss of the bandgap CP in Figure 5 and its replacement by a broad absorption onset typical of a disordered or nanocrystalline semiconductor. From the SEM images and XRD patterns, a strong reduction in grain size is observed between the available results for *x* = 0.31 and *x* = 0.56, which is consistent with the reduction in surface roughness in Figure 17.

## 4. Summary and Conclusions

In this study, a complex dielectric function (ε_1_, ε_2_) database has been developed for (In_1−x_Ga_x_)_2_Se_3_ (IGS) thin films deposited in a process that simulates the first stage of three-stage deposited Cu(In_1−x_Ga_x_)Se_2_ (CIGS) absorber layers of CIGS solar cells. This database is the first step in (i) understanding the deposition process and structure of IGS films with different Ga compositions and thicknesses and (ii) developing the capability of composition, thickness and structural monitoring and control during CIGS solar cell deposition. The (ε_1_, ε_2_) spectra of IGS determined within the 3500–4100 Å thickness range for the 9000 Å thick films studied here are found to exhibit different behaviors for *x* ≤ 0.45 and *x* > 0.56. For *x* ≤ 0.45, a sharp critical point (CP) resonance is observed that is characteristic of a large-grain polycrystalline structure and defines the fundamental bandgap of the IGS. For *x* ≥ 0.56, this well-defined CP feature disappears and is replaced by a gradual absorption onset with one broad resonance at high energies, characteristic of a highly disordered or nanocrystalline semiconductor. This transition in the (ε_1_, ε_2_) spectra represents a microstructural change in the IGS which remains hexagonal (defect-wurtzite) on both sides of the transition, as indicated by X-ray diffractometry. With an increase in *x* from 0.00 to 0.45, the CP bandgap energy is observed to increase monotonically with increasing *x* from ~2.19 to 2.33 eV. The width of this CP bandgap resonance exhibits a minimum at *x* ~ 0.20–0.30 suggesting the largest grain structure at this composition. The clear trends in the optical properties of the IGS, in particular, the monotonically increasing CP bandgap with increasing Ga composition over the range 0 ≤ *x* ≤ 0.45, suggest excellent prospects for real time monitoring and control of composition over this range. The capability of compositional monitoring is demonstrated through the analysis of in-situ SE data collected for a 1.7 μm thick IGS layer deposited in the first stage of a three-stage co-evaporation process that leads to a high efficiency CIGS solar cell.

The structural evolution of IGS films is found to depend sensitively on the Ga content *x*. For example, in the case of *x* = 0.00 (In_2_Se_3_), the surface is comparatively rough with a roughness layer thickness of ~160 Å at a bulk layer thickness of ~9000 Å. Thus, during film growth from a Mo surface whose roughness layer thickness is ~70 Å, In_2_Se_3_ growth leads to an enhancement in the roughness as compared to the substrate. In contrast, for *x* = 1.00 (Ga_2_Se_3_), the surface is comparatively smooth with a roughness layer thickness of ~40 Å at a bulk layer thickness of ~9000 Å. In this case, during film growth from a Mo surface whose roughness layer thickness is ~75 Å, Ga_2_Se_3_ growth leads to suppression in the roughness as compared to the substrate. The most rapid change in the final roughness thickness with composition is observed for between *x* = 0.45 and *x* = 0.56, likely due to the transition in the film microstructure without a change in crystal structure. For most *x* values, the surface roughness stabilizes versus time by the end of the deposition at a bulk layer thickness of ~9000 Å. Only for the depositions with *x* = 0.25 and 0.31, however, does the roughness thickness increase throughout deposition, indicating continuous growth of grains that protrude above the surface. In addition, for IGS films with compositions *x* in the range 0.25–0.37, the initial growth smoothening occurs at a faster rate and coalescence to a smooth surface occurs in shorter time than for IGS films in other compositional ranges. Both observations suggest enhanced surface transport of film precursors during IGS deposition with compositions near *x* ~ 0.3.

The behaviors of both the (ε_1_, ε_2_) spectra and the endpoint surface roughness evolution as a function of *x* are consistent with direct SEM observations of the morphology. SEM images show that the crystalline grain size increases from *x* = 0 to *x* = 0.31, consistent with the sharpening of the bandgap CP and the development of a long-term roughening trend with the increase in *x* over this range. The SEM images also show a rapid decrease in grain size between *x* = 0.31 and 0.56 which may be consistent with the suppression of the bandgap CP in the (ε_1_, ε_2_) spectra transitioning to a shape consistent with a more highly disordered or nanocrystalline structure. Thus, the SEM observations are also consistent with the much smoother surfaces deduced from RTSE results with increasing *x* above *x* = 0.56.

## Figures and Tables

**Table 1:** EDS results for the IGS films of the composition series. Additional columns tabulate the selected time ranges defined by the initial times t_i_ and the final times t_f_, as well as the inversion times (or center times) t*, used for multi-time analysis of RTSE data collected during the deposition of IGS with different compositions. This RTSE analysis is used to determine the (ε_1_, ε_2_) spectra of IGS, assuming a b-spline model, along with the evolution of the bulk and surface roughness layer thicknesses d_b_ and d_s_, respectively. The best fit results for d_b_ are given at t_i_, t* and t_f_ of the multi-time analysis; the best fit results for d_s_ and the fixed value of the void content f_v_ in the IGS surface roughness layer are given at t*; and the values describing the fitting quality are presented as the mean-square errors, MSE. The effective deposition rates, tabulated as the time derivatives of the effective thickness (IGS film volume/area) at t*, are provided in the last column. In most cases, these effective deposition rates are larger than (d_b_* + 0.5d_s_*)/t* since the latter average rate does not include the time-independent interface IGS contribution of d_i_ f_IGS_ and in addition, variations in rate can occur throughout the deposition.

EDS Measurement			Ga Content (*x*)	For Bulk Thickness of IGS at ~3500-4000 Å									d(d_eff_)/dt (Å/s)
In (at. %)	Ga (at. %)	Se (at. %)		t_i_ (min)	d_bi_ (Å)	t_f_ (min)	d_bf_ (Å)	t* (min)	d_b_* (Å)	d_s_* (Å)	f_v_* (vol.%)	MSE	
41.7	----	58.3	0.00	12.29	3437 ± 2	12.64	3542 ± 2	12.46	3489 ± 2	150 ± 5	50	10.7	5.05
40.2	4.6	55.2	0.10	8.86	3219 ± 2	10.09	3669 ± 2	9.53	3465 ± 2	95 ± 2	50	15.2	6.14
33.8	11.2	55.0	0.25	3.36	3095 ± 2	4.70	4386 ± 3	4.14	3842 ± 2	105 ± 1	50	20.3	16.14
28.9	13.1	58.0	0.31	5.25	3278 ± 2	6.52	4068 ± 2	5.83	3643 ± 2	90 ± 1	50	18.7	10.40
26.4	15.5	58.1	0.37	6.97	3527 ± 2	8.32	4245 ± 3	7.72	3924 ± 4	99 ± 1	50	16.8	8.88
25.3	20.4	54.3	0.45	4.15	3249 ± 2	5.58	4419 ± 3	4.84	3813 ± 2	103 ± 1	50	16.0	13.68
18.5	23.8	57.7	0.56	9.23	3679 ± 1	10.49	4229 ± 1	10.01	4019 ± 1	71 ± 1	50	8.64	7.28
12.3	27.5	60.2	0.69	12.14	3778 ± 1	13.61	4224 ± 1	13.05	4053 ± 1	55 ± 1	50	7.57	5.06
----	41.2	58.8	1.00	19.98	3908 ± 1	21.23	4171 ± 1	20.55	4025 ± 6	43 ± 2	50	6.67	3.51

**Table 2:** Expressions given in terms of IGS composition *x* with *x* ≤ 0.45 for the critical point (CP) and Tauc-Lorentz (TL) oscillator parameters that define the (ε_1_, ε_2_) spectra. Among the ten possible variable parameters of this oscillator model, three are fixed as shown and two are coupled in the fit. For the coupled parameters, the Tauc gap of the TL oscillator is equated to the fundamental bandgap, which is the resonance energy of the CP oscillator. This latter oscillator dominates the absorption onset over this range of *x*. The polynomial expressions are appropriate for an IGS temperature of 400 °C and a bulk layer thickness in the range of 3500–4100 Å.

Oscillator	Parameter	Expression in terms of *x*
CP	*A*_1_	− 1.335*x*^2^ + 1.597*x* + 2.314
	*E_g_*_1_ (eV)	0.4330*x*^2^ + 0.05997*x* + 2.190
	Γ_1_ (eV)	7.035*x*^2^ − 3.387*x* + 0.7913
	φ (degree)	0 (fixed)
	μ	0.5 (fixed)
TL	*A*_2_ (eV)	− 27.86*x*^2^ + 138.2*x* + 66.98
	*E_g_*_2_ (eV)	0.4330*x*^2^ + 0.05997*x* + 2.190
	Γ_2_ (eV)	1.186*x*^2^ + 4.531*x* + 2.445
	*E*_02_ (eV)	3.057*x*^2^ − 2.480*x* + 5.366
	ε_1,∞_	1 (fixed)

**Table 3:** Expressions given in terms of IGS composition *x* with *x* ≥ 0.56 for the critical point (CP) and Tauc-Lorentz (TL) oscillator parameters that define the (ε_1_, ε_2_) spectra. Among the ten possible variable parameters, four are fixed as shown and two are coupled in the fit. For the coupled parameters, the resonance energy of the very weak CP oscillator is equated to the Tauc gap of the TL oscillator which controls the absorption onset over this range of *x*. In fact, because of the low amplitude of the CP oscillator, its contribution to the spectra over this range is negligible. The polynomial expressions are appropriate for an IGS temperature of 400 °C and a bulk layer thickness in the range of 3500–4100 Å.

Oscillator	Parameter	Expression in terms of *x*
CP	*A*_1_	−1.309*x*^2^ + 1.981*x* − 0.6135
	*E_g_*_1_ (eV)	0.08500*x*^2^ + 0.4660*x* + 1.138
	Γ_1_ (eV)	0.4952*x*^2^+ 0.2431*x* + 1.106
	φ (degree)	0 (fixed)
	μ	0.5 (fixed)
TL	*A*_2_ (eV)	2.885*x*^2^ + 30.35*x* + 70.95
	*E_g_*_2_ (eV)	0.08500*x*^2^ + 0.4660*x* + 1.138
	Γ_2_ (eV)	1.916*x*^2^ − 4.019*x* + 7.041
	*E*_02_ (eV)	5.050 (fixed)
	ε_1,∞_	1 (fixed)

**Table 4:** Details of multi-time RTSE analysis that provides the structural evolution and b-spline (ε_1_, ε_2_) spectra for the IGS deposition with composition *x* = 0.30 on Mo-coated SLG. Selected initial, center and final times (t_i_, t* and t_f_) for multi-time analysis are provided along with the associated bulk layer thicknesses obtained in the analysis from the best overall fit. Also given from the analysis are the center time surface roughness layer thicknesses and the void volume percentages in the surface layer. The mean square error values in the best fit multi-time analysis are shown in the second-to-last column. The deposition rates, given as the time derivatives of the IGS effective thickness (film volume/area) at t*, are provided in the last column. These effective deposition rates are larger than (d_b_* + 0.5d_s_*)/t* since the latter average rate does not include the time-independent interface IGS contribution of d_i_ f_IGS_, and in addition, variations in rate can occur in the initial stages and throughout the deposition.

t_i_ (min)	d_bi_ (Å)	t_f_ (min)	d_bf_ (Å)	t* (min)	d_b_* (Å)	d_s_* (Å)	f_v_* (vol.%)	MSE	d(d_eff_)/dt (Å/s)
1.09	292 ± 2	1.32	432 ± 2	1.22	372 ± 2	49 ± 1	50	8.76	10.50
4.93	2616 ± 4	5.16	2754 ± 3	5.07	2695 ± 3	66 ± 1	50	11.1	10.04
6.40	3504 ± 2	6.76	3721 ± 2	6.60	3622 ± 2	81 ± 1	50	15.7	10.09
9.78	5380 ± 4	10.48	5792 ± 4	10.15	5599 ± 4	112 ± 1	44 ± 1	18.8	9.86
14.52	8890 ± 3	15.27	9377 ± 3	14.91	9144 ± 3	124 ± 1	46 ± 1	18.3	10.82

**Table 5:** Second-order polynomial expressions for the critical point (CP) and Tauc-Lorentz (TL) oscillator parameters of the (ε_1_, ε_2_) spectra for (In_1−x_Ga_x_)_2_Se_3_ with *x* = 0.30, given in terms of the bulk layer thickness d_b_. For all parameters with the exception of Г_2_ and *E*_02_, two different ranges of d_b_ are used as indicated for describing the polynomial. Among the ten possible variable parameters in an expression that combines CP and TL oscillators, three (φ, μ, ε_1,∞_) are fixed as shown and two (*E_g_*_1_, *E_g_*_2_) are coupled. Thus, the bandgap of the TL oscillator *E_g_*_2_ is equated to the fundamental bandgap *E_g_*_1_, which is the resonance energy of the CP oscillator. When the CP amplitude is strong, the CP oscillator controls the absorption onset; however, when the CP is very weak, the TL oscillator controls the onset.

Oscillator	Parameter	Expression in Terms of d_b_
CP	*A*_1_	0; 3 Å < d_b_ < 370 Å −2.114 × 10^−^^7^ d_b_^2^ + 14.40 × 10^−^^4^ d_b_ − 0.5056; 370 Å < d_b_ < 3600 Å −2.594 × 10^−^^8^ d_b_^2^ + 3.559 × 10^−^^4^ d_b_ + 1.041; d_b_ > 2700 Å
	*E_g_*_1_ (eV)	−1.065 × 10^−^^7^ d_b_^2^ + 7.959 × 10^−^^4^ d_b_ + 0.7296; 3 Å < d_b_ < 3600 Å −7.859 × 10^−^^9^ d_b_^2^ + 1.084 × 10^−^^4^ d_b_ + 1.847; d_b_ > 2700 Å
	Γ_1_ (eV)	−3.309 × 10^−^^8^ d_b_^2^ + 1.025 × 10^−^^4^ d_b_ + 0.5586; 3 Å < d_b_ < 3600 Å 1.148 × 10^−^^8^ d_b_^2^ − 1.577 × 10^−^^4^ d_b_ + 0.9371; d_b_ > 2700 Å
	φ (degree)	0 (fixed)
	μ	0.5 (fixed)
TL	*A*_2_ (eV)	−2.230 × 10^−^^6^ d_b_^2^ + 0.02542 d_b_ + 64.20; 3 Å < d_b_ < 3600 Å −1.233 × 10^−^^6^ d_b_^2^ + 0.011 d_b_ + 99.12; d_b_ > 2700 Å
	*E_g_*_2_ (eV)	−1.065 × 10^−^^7^ d_b_^2^ + 7.959 × 10^−^^4^ d_b_ + 0.7296; 3 Å < d_b_ < 3600 Å −7.859 × 10^−^^9^ d_b_^2^ + 1.084 × 10^−^^4^ d_b_ + 1.847; d_b_ > 2700 Å
	Γ_2_ (eV)	1.812 × 10^−^^8^ d_b_^2^ − 5.381 × 10^−^^4^ d_b_ + 5.987
	*E*_02_ (eV)	−2.695 × 10^−^^9^ d_b_^2^ + 5.646 × 10^−^^5^ d_b_ + 4.922
	ε_1,∞_	1 (fixed)

**Table 6:** Initial time t_i_, final time t_f_ and center time t*, selected for the multi-time analyses of RTSE data in the early stages of IGS depositions with different Ga contents *x* shown in the first column. The best fit bulk layer thicknesses at t_i_, t_f_, and t* are given, as well as the best fit surface roughness layer thickness and fixed void volume percentage in the surface roughness layer both at t*. The results were obtained from RTSE analysis which was also used to extract the early stage (ε_1_, ε_2_) spectra as b-splines. The mean square errors from the best fit analyses are shown in the second-to-last column. The deposition rates, provided in the last column, are given as the time derivative of the effective thickness (volume/area) at t*. These effective deposition rates are larger than (d_b_* + 0.5d_s_*)/t* since the latter average rate does not include the time-independent interface IGS contribution of d_i_ f_IGS_ and in addition, variations in rate can occur in the early stages of the deposition.

Ga Content (*x*)	For bulk thickness of IGS at ~ 350-410 Å									
	t_i_ (min)	d_bi_ (Å)	t_f_ (min)	d_bf_ (Å)	t* (min)	d_b_* (Å)	d_s_* (Å)	f_v_* (vol.%)	MSE	d(d_eff_)/dt (Å/s)
0.00	1.76	346 ± 4	2.06	425 ± 5	1.93	392 ± 4	8 ± 2	50	7.60	4.87
0.10	1.33	339 ± 4	1.67	453 ± 5	1.53	404 ± 3	7 ± 1	50	16.5	5.62
0.25	0.37	215 ± 1	0.66	476 ± 2	0.55	372 ± 1	4 ± 1	50	11.7	15.31
0.31	0.62	292 ± 2	0.93	488 ± 3	0.81	408 ± 2	8 ± 2	50	12.6	10.82
0.37	0.94	325 ± 5	1.18	441 ± 6	1.05	379 ± 4	11 ± 2	50	10.5	8.12
0.45	0.48	251 ± 4	0.79	483 ± 8	0.62	358 ± 3	8 ± 1	50	9.2	12.73
0.56	1.09	287 ± 1	1.69	522 ± 1	1.35	387 ± 1	32 ± 1	50	6.03	6.62
0.69	1.22	271 ± 1	1.83	462 ± 1	1.48	352 ± 1	21 ± 1	50	5.99	5.33
1.00	2.17	275 ± 1	2.78	401 ± 1	2.52	347 ± 1	22 ± 1	50	3.51	3.46

**Table 7:** Compositions, best fit mean square errors (MSE), along with fixed and variable parameters in the single Tauc-Lorentz (TL) oscillator expressions that define the (ε_1_, ε_2_) spectra at a measurement temperature of 400 °C for ~350–410 Å thick IGS films with different Ga contents *x*. One of the five possible variable parameters of the TL expression, ε_1,∞_, is fixed at 1 in the best fit analyses, as shown in the third column.

Ga Content (*x*)	MSE	ε_1__,∞_	For Bulk Thickness of IGS at ~350–410 Å			
			Tauc-Lorentz			
			*A*_2_ (eV)	Γ_2_ (eV)	*E*_02_ (eV)	*E_g_*_2_ (eV)
0.00	21	1	56.4 ± 2.9	5.08 ± 0.13	3.962 ± 0.059	0.961 ± 0.046
0.10	10	1	82.4 ± 2.4	6.16 ± 0.10	4.371 ± 0.038	1.111 ± 0.025
0.25	12	1	78.6 ± 1.6	5.72 ± 0.10	4.592 ± 0.021	1.156 ± 0.027
0.31	10	1	81.2 ± 2.3	5.94 ± 0.11	4.721 ± 0.033	1.111 ± 0.027
0.37	15	1	80.1 ± 2.1	6.13 ± 0.12	4.928 ± 0.025	1.154 ± 0.073
0.45	11	1	75.9 ± 2.3	5.34 ± 0.09	4.556 ± 0.032	1.119 ± 0.029
0.56	7	1	80.1 ± 1.6	5.41 ± 0.07	4.837 ± 0.021	1.279 ± 0.020
0.69	8	1	79.7 ± 1.8	5.27 ± 0.07	4.731 ± 0.024	1.229 ± 0.023
1.00	5	1	98.2 ± 1.7	5.20 ± 0.05	4.791 ± 0.019	1.580 ± 0.016
